# Association of interleukin-10 *rs1800896*, *rs1800872*, and interleukin-6 *rs1800795* polymorphisms with squamous cell carcinoma risk: A meta-analysis

**DOI:** 10.1515/biol-2022-0580

**Published:** 2023-04-15

**Authors:** Zhenxia Wei, Xiaoping Su, Qiurui Hu, Yonghui Huang, Cuiping Li, Xuanping Huang

**Affiliations:** Department of Oral and Maxillofacial Surgery, College & Hospital of Stomatology, Guangxi Medical University, Nanning 530021, PR China; Department of Experiment, College & Hospital of Stomatology, Guangxi Medical University, Nanning 530021, PR China; Guangxi Key Laboratory of Oral and Maxillofacial Rehabilitation and Reconstruction, Guangxi Clinical Research Center for Craniofacial Deformity, Guangxi Health Commission Key Laboratory of Prevention and Treatment for Oral Infectious Diseases, Nanning 530021, PR China; Department of Prosthodontics, College & Hospital of Stomatology, Guangxi Medical University, Nanning 530021, PR China

**Keywords:** squamous cell carcinoma, interleukin-10, interleukin-6, genetic polymorphism, meta-analysis

## Abstract

The relationship between interleukin (*IL*)-10 and *IL-6* gene polymorphisms and squamous cell carcinoma (SCC) has been demonstrated but with inconsistent conclusions. The aim of this study was to evaluate the potential associations of *IL* gene polymorphisms and the SCC risk. PubMed, Cochrane Library, Web of Science, China National Knowledge Infrastructure, China Biomedical Database, WanFang, and China Science and Technology Journal Database databases were searched for articles reporting the correlations of *IL-10* and *IL-6* gene polymorphisms with the SCC risk. Odds ratio and 95% confidence interval were calculated using Stata Version 11.2. Meta-regression, sensitivity, and publication bias were analyzed. False-positive reporting probability and Bayesian measure of the false-discovery probability were used to explore the credibility of the calculation. Twenty-three articles were included. The *IL-10 rs1800872* polymorphism showed a significant correlation with the SCC risk in the overall analysis. Studies pooled by ethnicity revealed that the *IL-10 rs1800872* polymorphism reduced the SCC risk in the Caucasian population. The results of this study suggest that the *IL-10 rs1800872* polymorphism may confer a genetic susceptibility to SCC, particularly oral SCC, in Caucasians. However, the *IL-10 rs1800896* or *IL-6 rs1800795* polymorphism was not significantly associated with the SCC risk.

## 1 Introduction

Cancer is a leading cause of death worldwide. According to the 2019 World Health Organization estimates, cancer was the leading cause of death in 112 of 183 countries and may surpass cardiovascular disease as the leading cause of death in many countries [1]. Squamous cell carcinoma (SCC) is a malignant tumor that arises in tissues and organs covered by the squamous epithelium, including the skin, oral cavity, esophagus, cervix, vagina, bronchus, urinary bladder, and renal pelvis [2–4]. Currently, combination therapy is the most common treatment modality for SCC [5,6]. Regardless of the treatment modality, patients develop severe scarring, increasing their financial burden and mental stress [7–9]. Extensive epidemiological and molecular biology studies have shown that chronic inflammation, unhealthy lifestyle, viral infections, and many other risk factors increase the SCC risk [10–12]; however, the specific role of these factors in tumor development has not been elucidated.

Processes such as inflammatory cell infiltration and malignant cell metastasis are common in cancer. Cytokines might play a critical role in these processes. Interleukin (IL) is an immunomodulatory cytokine involved in cell proliferation and apoptosis that can promote tumor immune escape and accelerate the progression of malignant tumors by inhibiting the anti-tumor immune response in the tumor microenvironment [13]. T-helper cytokines are utilized by IL-10 to regulate the growth and differentiation of natural immune cells, keratinocytes, and endothelial cells and inhibit the activation and effector functions of T cells [14]. IL-6 can participate in the differentiation regulation of B cells and promote the release of antibodies by B cells. It can be produced and secreted by tumor cells, involved in the proliferation and differentiation of malignant tumor cells, and expressed at high levels in serum and tumor tissues of most cancers [15]. *IL-10* and *IL-6* genes are located on chromosomes 10 and 7, respectively, and polymorphic in the region where the gene begins transcription. In recent years, the relationship between *IL-10* and *IL-6* gene polymorphisms and cancer have attracted great attention. Gene polymorphisms are closely related to changes in the IL expression level, leading to the occurrence of many cancers. Gene polymorphisms in the *IL-10* and *IL-6* promoter regions might affect the expression of gene-encoded proteins associated with the risk and prognosis of SCC [16,17]. The correlations of *IL-10 rs1800896(-1082)* and *rs1800872(-592)* and *IL-6 rs1800795(-174)* promoter-region polymorphisms with the SCC risk have been extensively studied. The polymorphisms are located near the transcription factor binding site and related to the pathogenesis of SCC, including cervical SCC [18]. However, the study results have been inconclusive and inconsistent [19,20].

The advantage of meta-analyses is reduction in random errors through quantitative and comprehensive analyses of all eligible research data. To date, no studies have focused on the correlations of *IL-10* or *IL-6* gene polymorphisms with the SCC risk. Therefore, the aim of this meta-analysis was to clarify the correlations of the *IL-10 rs1800896* and *rs1800872* and *IL-6 rs1800795* gene polymorphisms with the SCC risk, including subgroup analyses by ethnicity, control source, and cancer type. The risk assessment was expected to be more detailed and accurate compared to previous studies. At the same time to provide ideas for cancer prevention and clinical treatment.

## 2 Materials and methods

### 2.1 Literature search

We extracted articles reporting the correlations of *IL-10 rs1800896* and *rs1800872* and *IL-6 rs1800795* gene polymorphisms with the SCC risk from PubMed, Cochrane Library, Web of Science, China National Knowledge Infrastructure, China Biomedical Database, WanFang, and China Science and Technology Journal Database databases. The search terms were (“Interleukin-10” OR “IL-10” OR “IL10”) (“Interleukin-6” OR “IL-6” OR “IL6”) AND (“squamous cell carcinoma” OR “squamous cancer” OR “squamous cell tumor”) AND (“polymorphism” OR “genetic polymorphism” OR “polymorphisms”). Moreover, references of relevant studies, including meta-analyses, were searched manually to screen more studies for inclusion. We limited our search to human studies and did not specify any minimum number of cases or controls required or the year of publication. Articles published in English or Chinese were likely to be included.

### 2.2 Inclusion and exclusion criteria

Inclusion criteria were (1) case–control study reporting the associations of *rs1800896*, *rs1800872*, and *rs1800795* polymorphisms in the promoter regions of *IL-10* and *IL-6* genes with the SCC risk, (2) information available regarding the distribution of cases and controls, allowing calculation of the odds ratio (OR) with 95% confidence interval (CI), and (3) full text available without duplication. Exclusion criteria were (1) original study design other than case–control or study without genotype data, (2) cancer not specified as SCC, (3) case report, (4) non-human study, (5) review (including meta-analysis), and (6) duplicate or overlapping data.

### 2.3 Study design and extracted information

We extracted the following information: surname of the first author, date of publication, country, participants’ ethnicity (Asian, Caucasian, or mixed descent), control source (population-, or hospital-based), cancer type (e.g., oral SCC), sample sizes of case and control groups, and detailed data on the genetics and genotyping of case and control studies. Two investigators (Z.W. and X.S.) independently extracted information based on the constituted standards, and a third investigator (Q.H.) reviewed the information. Disagreements were resolved through a discussion among the three investigators, ensuring more accurate data extraction. Investigators selected studies by reviewing the abstracts and full text based on the aforementioned eligibility criteria. We manually searched the references in selected studies, including meta-analyses, to screen more studies for inclusion. Among similar studies, those with the largest sample size or most recent publication were selected.

### 2.4 Statistical analysis

The correlations of *IL-10 rs1800896* and *rs1800872* and *IL-6 rs1800795* gene polymorphisms with the SCC risk were estimated using 95% CI and OR. The significance of OR was determined with the *z*-test. A *p*-value <0.05 was set to indicate statistical significance. The combined OR was evaluated using four genetic models, including homozygote comparison (GG/AA; CC/AA; CC/GG), heterozygote comparison (AG/AA; AC/AA; GC/GG), dominant (AG + GG/AA; AC + CC/AA; GC + CC/GG), and recessive (GG/AA + AG; CC/AA + AC; CC/GG + GC) models in *IL-10 rs1800896* and *rs1800872* and *IL-6 rs1800795*. Subgroup analyses were conducted by cancer type, ethnicity, and control source. *I* ^2^ statistics were used to evaluate the heterogeneity among studies. Studies were homogenous at *I* ^2^ < 50%, and the fixed-effects model was used to combine the OR and 95% CI; otherwise, the random-effects model was used.

The occurrence of publication bias was determined with Egger’s and Begg’s tests. Stata version 11.2 (Stata Corporation, College Station, TX) was used, with the significance level set as a bilateral *α* of 0.05. A *p*-value <0.05 was considered to indicate publication bias.

Three predefined sources of heterogeneity were detected using meta-regression analyses: publication year, ethnicity, and control source. The rationality of the meta-analysis results was checked with sensitivity analyses. Excluding one study each time and combining the remaining studies revealed no substantial changes in the corresponding combined OR; thus, our results were considered to be statistically robust. In addition, the false-positive reporting probability (FPRP) was evaluated. We confirmed a FPRP <0.2 and appointed prior probabilities of 0.25, 0.1, 0.01, 0.001, and 0.0001 to examine an OR of 1.5 associated with the SCC risk. The results were significant at FPRP <0.2 [21]. The Bayesian measure of false-discovery probability (BFDP) was estimated using Excel computed tables to evaluate the reliability of the statistically significant associations [22]. The results were significant at BFDP <0.8.

## 3 Results

### 3.1 Study characteristics

**Figure 1:**
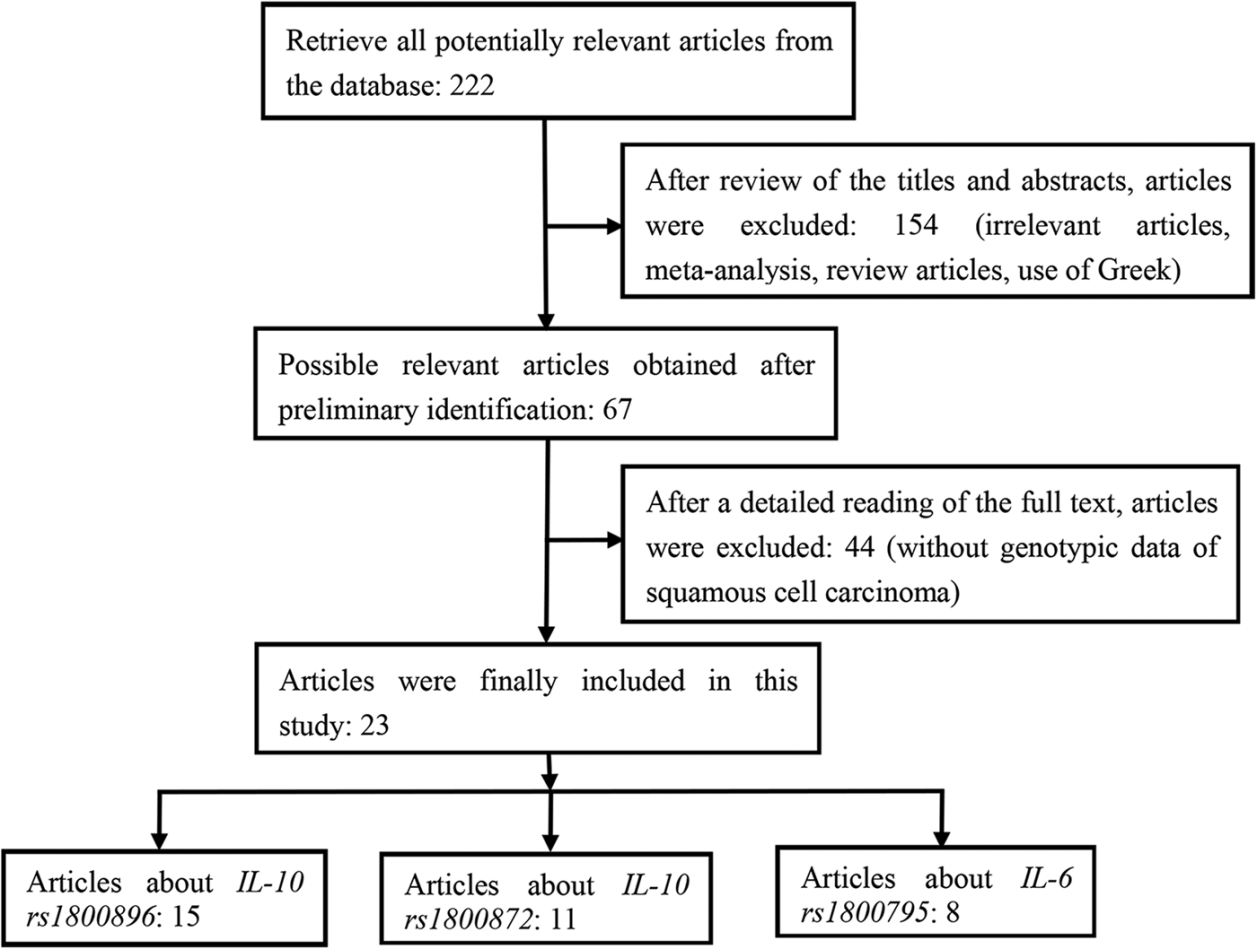
Whole flow diagram of the study selection process.

After applying the eligibility criteria, 222 articles were retrieved. After reading the titles and abstracts, 154 articles were excluded for reasons such as irrelevant study topic, meta-analysis or review design, and Greek language. After reviewing the full text of the remaining 67 articles, 44 articles without genotypic data of patients with SCC were excluded. Finally, 23 articles met all requirements for inclusion in this study. Figure 1 shows a flowchart of the study selection process.

Fifteen studies, involving 3,311 cases and 4,756 controls, investigated the association of the *IL-10 rs1800896* polymorphism with the SCC risk [16,19,20,23–34]. Eleven studies, involving 3,069 cases and 4,265 controls, investigated the association of the *IL-10 rs1800872* polymorphism with the SCC risk [16,20,24,26,27,29,30,34–37]. Eight studies, involving 1,315 cases and 1,905 controls, investigated the association of the *IL-6 rs1800795* polymorphism with the SCC risk [20,33,38–43]. Nine and six of the 15 included studies on *IL-10 rs1800896* involved the Caucasian and Asian ethnicities, respectively. Control sources were hospital-based in six studies and population-based in nine studies. A total of six cancer types were reported. Oral and cervical SCC were reported in five and three articles, respectively. Lung, head and neck, laryngeal, and esophageal SCC were reported in one article each. Table 1 shows the characteristics of the included studies on *IL-10 rs1800896*.

**Table 1:** Characteristics of included case–control studies on *IL-10 rs1800896* polymorphism and squamous cell carcinoma

No.	Author	Year	Ethnicity	Country	Cancer type	Source of control	Sample size of case	Sample size of control	Genotype distribution						MAF	Genotyping method
									Case			Control				
									AA	AG	GG	AA	AG	GG		
1	Pasvenskaite et al.	2021	Caucsian	Lithuania	Laryngeal SCC	HB	300	533	70	163	67	148	269	116	0.48	PCR
2	Mao et al.	2021	Asian	China	Oral SCC	HB	125	110	109	16	0	98	12	0	0.06	PCR
3	Chen et al.	2020	Asian	China	Esophageal SCC	HB	721	1,208	625	84	4	1,061	136	4	0.06	PCR
4	Goud et al.	2019	Asian	Malaysia	Oral SCC	PB	41	48	37	4	0	39	9	0	0.07	PCR-RFLP
5	Sharma et al.	2018	Caucsian	India	Oral SCC	PB	100	150	51	36	13	100	50	0	0.22	PCR
6	Hussain et al.	2016	Caucsian	India	Oral SCC	HB	232	221	69	158	5	127	93	1	0.29	PCR-RFLP
7	Torres-Poveda et al.	2016	Caucsian	Mexico	Cervical SCC	PB	200	200	121	70	9	110	78	12	0.24	PCR
8	Zhou et al.	2014	Asian	China	Laryngeal SCC	PB	146	119	115	26	5	107	11	1	0.09	PCR-RFLP
9	Torres-Poveda et al.	2012	Caucsian	Mexico	Cervical SCC	HB	204	166	125	66	13	92	62	12	0.24	PCR
10	Jeong et al.	2010	Asian	Korea	Head and neck SCC	HB	290	358	238	38	2	304	45	1	0.07	PCR
11	Vairaktaris et al.	2008	Caucsian	Greece and Germany	Oral SCC	PB	144	141	46	96	2	81	60	0	0.28	PCR
12	Guo et al.	2005	Asian	China	Esophageal SCC	PB	203	443	117	81	5	267	164	12	0.22	PCR-RFLP
13	Zoodsma et al.	2005	Caucsian	Holland	Cervical SCC	PB	512	606	121	242	149	130	307	169	0.47	PCR
14	Seifart et al.	2005	Caucsian	Germany	Lung SCC	PB	40	243	13	17	10	86	115	42	0.42	PCR
15	El-Omar et al.	2003	Caucsian	United States	Esophageal SCC	PB	53	210	16	28	9	59	103	48	0.47	PCR-TaqMan

Abbreviations: PB, population-based; HB, hospital-based; SCC, squamous cell carcinoma; MAF, minor allele frequency.

Seven and four of the 11 included studies on *IL-10 rs1800872* involved the Caucasian and Asian populations, respectively. Control sources were population-based in six studies and hospital-based in five studies. A total of four cancer types were reported. Esophageal, cervical, oral, and lung SCC were reported in four, three, two, and two articles, respectively. Table 2 shows the characteristics of the included studies on *IL-10 rs1800872*.

**Table 2:** Characteristics of included case–control studies on *IL-10 rs1800872* polymorphism and squamous cell carcinoma

No.	Author	Year	Ethnicity	Country	Cancer type	Source of control	Sample size of case	Sample size of control	Genotype distribution						MAF	Genotyping method
									Case			Control				
									AA	AC	CC	AA	AC	CC		
1	Pasvenskaite et al.	2021	Caucsian	Lithuania	Laryngeal SCC	HB	300	533	21	102	177	33	179	321	0.23	PCR
2	Chen et al.	2020	Asian	China	Esophageal SCC	HB	721	1,208	349	301	65	550	523	128	0.31	PCR
3	Sharma et al.	2018	Caucsian	India	Oral SCC	PB	100	150	25	54	21	18	88	44	0.47	PCR
4	Torres-Poveda et al.	2016	Caucsian	Mexico	Cervical SCC	HB	200	200	58	98	44	30	85	85	0.47	PCR
5	Singh et al.	2017	Caucsian	India	Oral SCC	PB	250	250	39	168	43	14	173	63	0.45	PCR-RFLP
6	Zhou et al.	2014	Asian	China	Laryngeal SCC	PB	146	119	63	70	13	64	39	16	0.32	PCR-RFLP
7	Sun et al.	2013	Asian	China	Esophageal SCC	HB	380	380	162	163	31	191	141	33	0.28	PCR
8	Torres-Poveda et al.	2012	Caucsian	Mexico	Cervical SCC	HB	204	166	49	105	50	30	70	66	0.45	PCR
9	Wang et al.	2006	Asian	China	Esophageal SCC	PB	203	443	95	88	20	182	196	65	0.35	PCR-RFLP
10	Zoodsma et al.	2005	Caucsian	Holland	Cervical SCC	PB	512	606	25	172	300	26	175	405	0.20	PCR
11	El-Omar et al.	2003	Caucsian	United States	Esophageal SCC	PB	53	210	3	15	35	13	70	127	0.22	PCR-TaqMan

Abbreviations: PB, population-based; HB, hospital-based; SCC, squamous cell carcinoma; MAF, minor allele frequency.

Six and two of the nine included studies on *IL-6 rs1800795* involved the Caucasian and Asian populations, respectively. Control sources were population-based in six studies and hospital-based in two studies. A total of five cancer types were reported. Oral and laryngeal cancer were reported in three and two articles, respectively. Lung, cervical, and esophageal SCC were reported in article each. Table 3 shows the characteristics of the included studies on *IL-6 rs1800795*.

**Table 3:** Characteristics of included case–control studies on *IL-10 rs1800795* polymorphism and squamous cell carcinoma

No.	Author	Year	Ethnicity	Country	Cancer type	Source of control	Sample size of case	Sample size of control	Genotype distribution						MAF	Genotyping method
									Case			Control				
									GG	GC	CC	GG	GC	CC		
1	Pasvenskaite et al.	2020	Caucsian	Lithuania	Laryngeal SCC	HB	352	538	69	182	102	132	261	145	0.47	PCR
2	Candan Demiröz Abakay	2020	Caucsian	Turkey	Laryngeal SCC	PB	80	50	38	31	11	29	15	5	0.30	PCR
3	Fernández-Mateos et al.	2019	Caucsian	Spanish	Oral SCC	PB	70	70	12	33	25	8	23	39	0.34	PCR
4	Shi et al.	2014	Asian	China	Cervical SCC	HB	418	518	131	201	86	181	259	78	0.42	PCR-RFLP
5	Gaur et al.	2011	Asian	India	Oral SCC	PB	140	120	98	35	7	65	41	14	0.23	PCR-RFLP
6	Vairaktaris et al.	2008	Caucsian	Greece and Germany	Oral SCC	PB	162	156	42	102	18	90	60	6	0.33	PCR
7	Seifart et al.	2005	Caucsian	Germany	Lung SCC	PB	40	243	17	19	4	90	107	46	0.40	PCR
8	El-Omar et al.	2003	Caucsian	United States	Esophageal SCC	PB	53	210	13	6	5	83	98	28	0.32	PCR-TaqMan

Abbreviations: PB, population-based; HB, hospital-based; SCC, squamous cell carcinoma; MAF, minor allele frequency.

### 3.2 Quantitative synthesis

**Figure 2:**
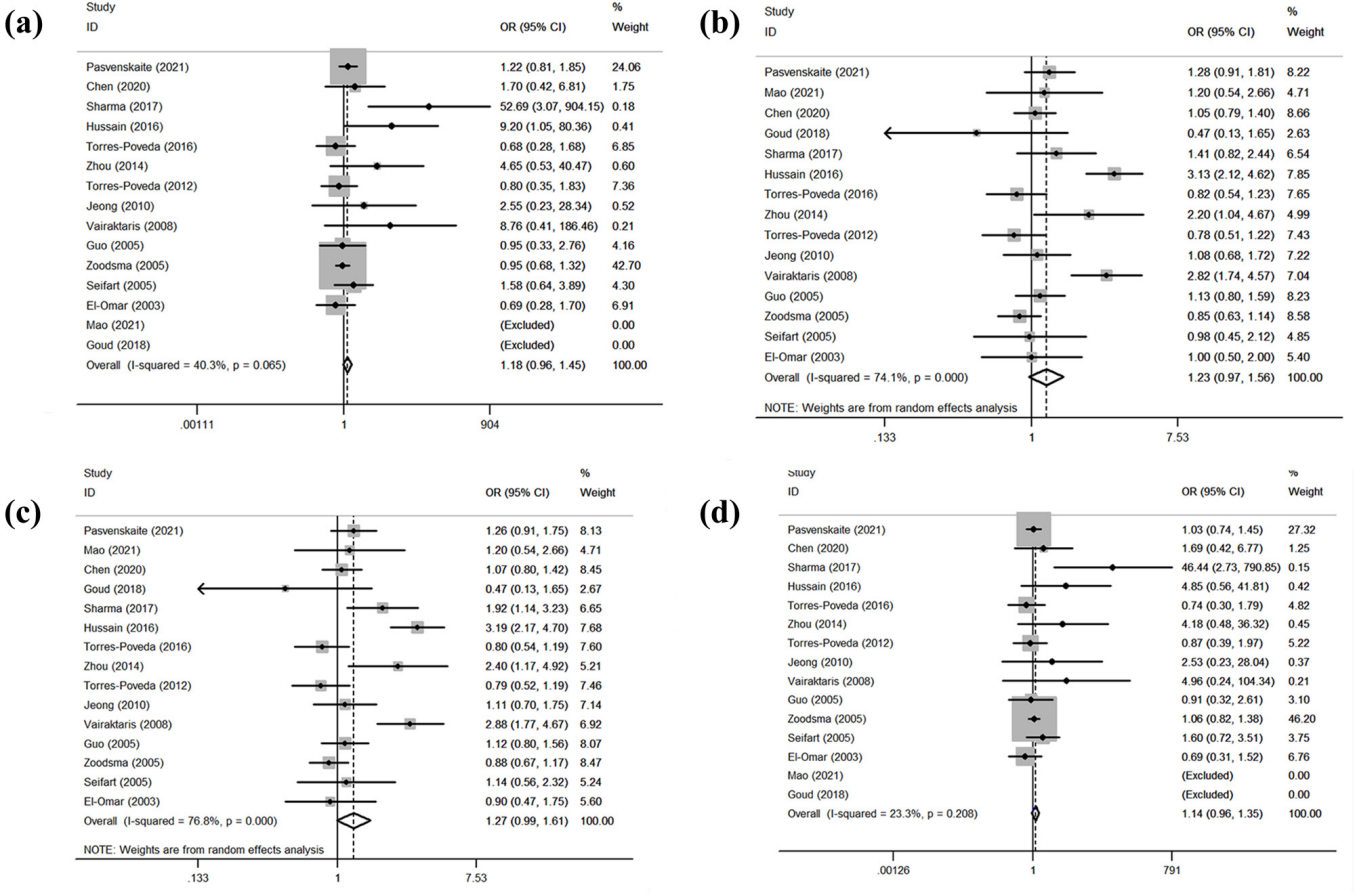
Statistical relationship between *IL-10 rs1800896* gene polymorphism and squamous cell carcinoma susceptibility in four models: (a) GG vs AA, (b) AG vs AA, (c) AG + GG vs AA, and (d) GG vs AA + AG. Abbreviations: OR: odds ratio; CI: confidence interval.

Table 4 shows the relationship between the *IL-10 rs1800896* gene polymorphism and the SCC risk. Data of 15 studies combined and analyzed according to four models revealed no significant association between the *IL-10 rs1800896* gene polymorphism and the SCC risk (GG/AA: OR = 1.18, 95% CI: 0.96–1.45, *p* = 0.109; AG/AA: OR = 1.27, 95% CI: 0.99–1.61, *p* = 0.057; AG + GG/AA: OR = 1.27, 95% CI: 0.99–1.61, *p* = 0.057; GG/AA + AG: OR = 1.14, 95% CI: 0.96–1.35, *p* = 0.149; Figure 2). Subgroup analyses by ethnicity and control source revealed no significant correlation between the *IL-10 rs800896* gene polymorphism and the SCC risk in any model. The subgroup analysis by cancer type revealed that the *IL-10 rs1800896* gene polymorphism significantly increased the oral SCC risk in all four models (GG/AA: OR = 19.09, 95% CI: 4.48–81.45, *p* = 0.000; AG/AA: OR = 1.76, 95% CI: 1.05–2.95, *p* = 0.045; AG + GG/AA: OR = 1.94, 95% CI: 1.21–3.13, *p* = 0.006; GG/AA + AG: OR = 12.70, 95% CI: 3.09–52.16, *p* = 0.000). In addition, the *IL-10 rs1800896* gene polymorphism increased the risk of laryngeal SCC (AG/AA: OR = 1.41, 95% CI: 1.04–1.93, *p* = 0.030), esophageal SCC (AG + GG/AA: OR = 0.48, 95% CI: 0.32–0.72, *p* = 0.000). Two gene models (AG/AA: *I* ^2^ = 74.10%, *p* = 0.000; AG + GG/AA: *I* ^2^ = 76.80%, *p* = 0.000). No positive results were found other than those aforementioned.

**Table 4:** Stratified analyses of the *IL-10 rs1800896* polymorphism on squamous cell carcinoma risk

Comparative model	No.	*Z*	*p*	OR (95% CI)	Heterogeneity			*Z*	Begg's test	*t*	Egger's test	FPRP *p*-value	FPRP statistical power	FPRP prior probability					BEDP prior probability		
					Heterogeneity chi-squared	*p*	*I* ^2^							0.25	0.10	0.01	0.001	0.0001	0.010	0.001	0.000
**GG/AA**																					
Overall	15	1.60	0.109	1.182(0.963–1.451)	20.09	0.065	40.30%	2.99	0.003	2.78	0.018	**0.110**	0.989	0.250	0.500	0.917	0.991	0.999	0.994	0.999	1.000
**Ethnicity**																					
Caucasian	9	0.72	0.470	1.166(0.769–1.769)	17.35	0.027	53.90%	1.56	0.118	2.04	0.080	0.470	0.882	0.615	0.828	0.981	0.998	1.000	0.996	1.000	1.000
Asian	6	1.25	0.212	1.571(0.773–3.191)	1.99	0.575	0.00%	1.02	0.308	3.09	0.091	0.212	0.449	0.586	0.809	0.979	0.998	1.000	0.991	0.999	1.000
**Source of control**																					
HB	6	1.36	0.175	1.269(0.899–1.792)	4.94	0.293	19.10%	1.22	0.221	1.33	0.276	**0.176**	0.829	0.389	0.657	0.955	0.995	1.000	0.993	0.999	1.000
PB	9	0.71	0.477	1.214(0.711–2.073)	14.52	0.043	51.80%	1.86	0.063	2.21	0.069	0.477	0.781	0.647	0.846	0.984	0.998	1.000	0.995	0.999	1.000
**Cancer types**																					
Laryngeal SCC	2	1.29	0.195	1.305(0.872–1.951)	1.43	0.233	29.80%	0.00	1.000	.	.	**0.194**	0.751	0.437	0.700	0.962	0.996	1.000	0.993	0.999	1.000
Oral SCC	5	3.98	0.000	19.093(4.475–81.451)	1.17	0.556	0.00%	0.00	1.000	0.44	0.734	**0.000**	**0.000**	0.408	0.674	0.958	0.996	1.000	0.873	0.986	1.000
Cervical SCC	3	0.74	0.459	0.896(0.670–1.199)	0.54	0.765	0.00%	1.04	0.296	−3.59	0.173	0.460	0.977	0.586	0.809	0.979	0.998	1.000	0.997	1.000	1.000
Esophageal SCC	3	0.29	0.771	0.913(0.495–1.686)	1.14	0.566	0.00%	1.04	0.296	44.50	0.014	0.771	0.842	0.733	0.889	0.989	0.999	1.000	0.995	0.999	1.000
**Genotyping method**																					
PCR	10	1.42	0.157	1.171(0.941–1.458)	13.47	0.097	40.60%	2.19	0.029	2.240	0.06	**0.158**	0.987	0.325	0.591	0.941	0.994	0.999	0.995	0.999	1.000
PCR-RFLP	4	1.29	0.199	2.670(0.597–11.936)	4.38	0.112	54.30%	1.04	0.296	4.690	0.134	**0.199**	0.225	0.726	0.888	0.989	0.999	1.000	0.989	0.999	1.000
**AG/AA**																					
Overall	15	1.68	0.094	1.226(0.990–1.556)	54.14	0.000	74.10%	0.40	0.692	0.27	0.792	**0.094**	0.951	0.228	0.470	0.907	0.990	0.999	0.992	0.999	1.000
**Ethnicity**																					
Caucasian	9	1.31	0.190	1.277(0.886–1.840)	48.04	0.000	83.30%	0.31	0.754	0.28	0.790	**0.189**	0.806	0.414	0.679	0.959	0.996	1.000	0.993	0.999	1.000
Asian	6	1.17	0.243	1.116(0.928–1.342)	5.18	0.395	3.40%	0.00	1.000	0.11	0.921	0.243	0.999	0.422	0.687	0.960	0.996	1.000	0.997	1.000	1.000
**Source of control**																					
HB	6	1.23	0.220	1.282(0.862–1.905)	27.41	0.000	81.80%	0.00	1.000	0.01	0.991	0.219	0.781	0.457	0.716	0.965	0.996	1.000	0.993	0.999	1.000
PB	9	1.06	0.291	1.183(0.866–1.616)	25.35	0.001	68.40%	0.10	0.917	0.49	0.638	0.291	0.932	0.484	0.737	0.969	0.997	1.000	0.995	1.000	1.000
**Cancer types**																					
Laryngeal SCC	2	2.17	0.030	1.414(1.035–1.931)	1.64	0.201	39.00%	0.00	1.000	.	.	**0.029**	0.645	**0.120**	0.291	0.818	0.978	0.998	0.976	0.998	1.000
Oral SCC	5	2.12	0.034	1.757(1.045–2.954)	14.70	0.005	72.80%	1.71	0.086	−3.83	0.031	**0.033**	0.275	0.267	0.523	0.923	0.992	0.999	0.973	0.997	1.000
Cervical SCC	3	1.79	0.073	0.823(0.666–1.018)	0.09	0.958	0.00%	1.04	0.296	−2.96	0.207	**0.073**	0.974	0.183	0.401	0.881	0.987	0.999	0.991	0.999	1.000
Esophageal SCC	3	0.65	0.513	1.073(0.869–1.325)	0.14	0.932	0.00%	0.00	1.000	−0.36	0.782	0.513	0.999	0.606	0.822	0.981	0.988	1.000	0.998	1.000	1.000
**Genotyping method**																					
PCR	10	0.98	0.325	1.119(0.894–1.400)	23.43	0.005	61.60%	0.89	0.371	0.79	0.455	0.325	0.995	0.495	0.746	0.970	0.997	1.000	0.997	1.000	1.000
PCR-RFLP	4	1.22	0.222	1.555(0.766–3.158)	19.37	0.000	84.50%	−0.34	1.000	−0.3	0.791	0.221	0.460	0.591	0.813	0.979	0.998	1.000	0.991	0.999	1.000
**AG+GG/AA**																					
Overall	15	1.91	0.057	1.266(0.993–1.614)	60.24	0.000	76.80%	0.99	0.322	0.51	0.619	**0.057**	0.914	**0.157**	0.359	0.860	0.984	0.998	0.988	0.999	1.000
**Ethnicity**																					
Caucasian	9	1.51	0.131	1.328(0.919–1.917)	53.02	0.000	84.90%	0.52	0.602	0.58	0.582	**0.130**	0.742	0.344	0.612	0.945	0.994	0.999	0.991	0.999	1.000
Asian	6	1.40	0.162	1.138(0.950–1.363)	6.33	0.276	21.00%	0.38	0.707	0.16	0.879	**0.160**	0.999	0.325	0.591	0.941	0.994	0.999	0.996	1.000	1.000
**Source of control**																					
HB	6	1.27	0.204	1.291(0.870–1.916)	28.60	0.000	82.50%	0.00	1.000	0.08	0.940	0.205	0.772	0.443	0.705	0.963	0.996	1.000	0.993	0.999	1.000
PB	9	1.31	0.190	1.266(0.993–1.614)	30.76	0.000	74.00%	0.73	0.466	0.67	0.523	**0.057**	0.914	0.157	0.359	0.860	0.984	0.998	0.988	0.999	1.000
**Cancer types**																					
Laryngeal SCC	2	0.25	0.802	1.202(0.286–5.053)	12.77	0.000	92.20%	0.00	1.000	.	.	0.802	0.619	0.795	0.921	0.992	0.999	1.000	0.992	0.999	1.000
Oral SCC	5	2.42	0.016	1.374(1.062–1.777)	6.12	0.191	34.60%	1.71	0.086	−2.21	0.114	**0.015**	0.748	**0.058**	**0.157**	0.672	0.954	0.995	0.964	0.996	1.000
Cervical SCC	3	1.12	0.263	0.892(0.730–1.090)	1.09	0.579	0.00%	1.04	0.296	1.31	0.416	0.264	0.998	0.442	0.704	0.963	0.996	1.000	0.997	1.000	1.000
Esophageal SCC	3	3.58	0.000	0.482(0.323–0.719)	5.29	0.071	62.20%	1.04	0.296	−4.80	0.131	**0.000**	**0.056**	**0.018**	**0.053**	0.381	0.861	0.984	**0.505**	0.911	0.999
**Genotyping method**																					
PCR	10	1.32	0.187	1.171(0.926–1.481)	27.73	0.001	67.50%	1.79	0.074	1.11	0.3	**0.187**	0.981	0.365	0.633	0.950	0.995	0.999	0.995	1.000	1.000
PCR-RFLP	4	1.26	0.209	1.591(0.772–3.280)	21.03	0.000	85.70%	−0.34	1.000	−0.23	0.838	0.208	0.437	0.589	0.811	0.979	0.998	1.000	0.990	0.999	1.000
**GG/AA+AG**																					
Overall	15	1.44	0.149	1.136(0.955–1.352)	15.65	0.208	23.30%	2.01	0.044	2.37	0.037	**0.151**	0.999	0.312	0.576	0.937	0.993	0.999	0.996	1.000	1.000
**Ethnicity**																					
Caucasian	9	1.21	0.228	1.117(0.933–1.336)	13.10	0.109	38.90%	0.94	0.348	1.66	0.140	0.226	0.999	0.404	0.670	0.957	0.996	1.000	0.997	1.000	1.000
Asian	6	1.12	0.263	1.496(0.739–3.031)	1.94	0.584	0.00%	1.02	0.308	3.23	0.084	0.264	0.503	0.611	0.825	0.981	0.998	1.000	0.992	0.999	1.000
**Source of control**																					
HB	6	0.60	0.548	1.096(0.813–1.476)	3.08	0.545	0.00%	1.22	0.221	2.15	0.121	0.546	0.981	0.626	0.834	0.982	0.998	1.000	0.997	1.000	1.000
PB	9	1.34	0.179	1.158(0.935–1.434)	12.67	0.081	44.70%	1.36	0.174	1.50	0.184	**0.179**	0.991	0.351	0.619	0.947	0.994	0.999	0.995	1.000	1.000
**Cancer types**																					
Laryngeal SCC	2	0.48	0.633	1.085(0.777–1.514)	1.58	0.209	36.50%	0.00	1.000	.	.	0.631	0.972	0.661	0.854	0.985	0.998	1.000	0.997	1.000	1.000
Oral SCC	5	3.53	0.000	12.702(3.093–52.156)	1.94	0.380	0.00%	0.00	1.000	0.49	0.709	**0.000**	**0.002**	0.454	0.714	0.965	0.996	1.000	0.923	0.992	1.000
Cervical SCC	3	0.13	0.895	1.016(0.800–1.290)	0.74	0.691	0.00%	1.04	0.296	−3.83	0.163	0.896	0.999	0.729	0.890	0.989	0.999	1.000	0.998	1.000	1.000
Esophageal SCC	3	0.51	0.610	0.863(0.489–1.521)	1.21	0.545	0.00%	1.04	0.296	5.60	0.112	0.610	0.814	0.692	0.871	0.987	0.999	1.000	0.995	0.999	1.000
**Genotyping method**																					
PCR	10	1.45	0.148	1.145(0.953–1.376)	1.88	0.209	26.50%	1.15	0.251	2.13	0.071	**0.148**	0.998	0.309	0.573	0.937	0.993	0.999	0.995	1.000	1.000
PCR-RFLP	4	1.32	0.18	1.696(0.774–3.717)	2.93	0.231	31.70%	0.00	1.000	16.96	0.037	**0.186**	0.380	0.596	0.816	0.980	0.998	1.000	0.990	0.999	1.000

Abbreviations: OR, odds ratio; CI, confidence interval; PB, population-based; HB, hospital-based; SCC, squamous cell carcinoma; FPRP, false positive report probability; BFDP, Bayesian false discovery probability. The results in bold represented that there was statistically significant noteworthiness at 0.2 level by FPRP or 0.8 level by BFDP calculations.

**Figure 3:**
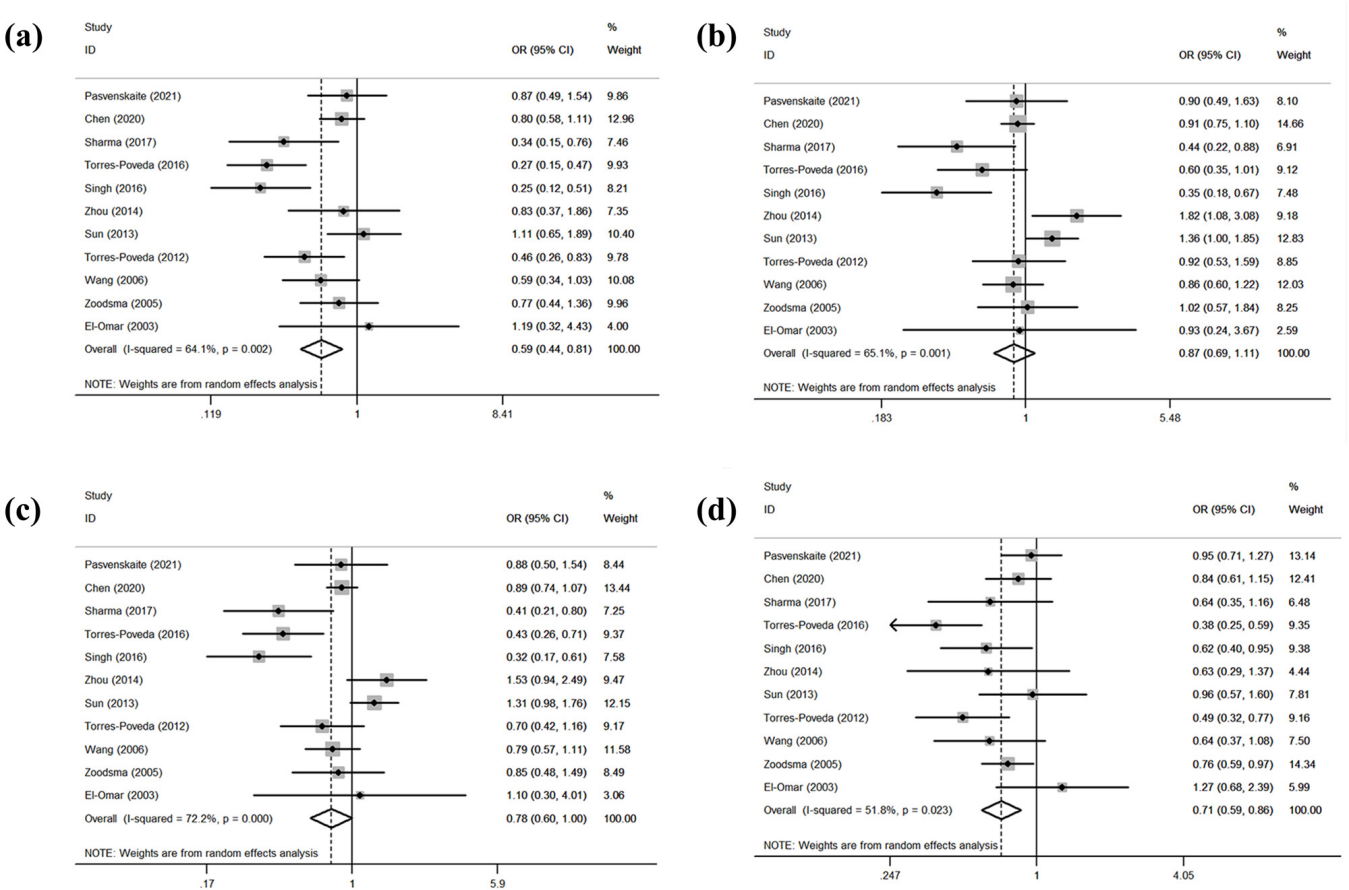
Statistical relationship between *IL-10 rs1800872* gene polymorphism and squamous cell carcinoma susceptibility in four models: (a) CC vs AA, (b) AC vs AA, (c) AC + CC vs AA, and (d) CC vs AA + AC.

Table 5 summarizes the relationship between the *IL-10 rs1800872* gene polymorphism and the SCC risk. The overall analysis revealed that the *IL-10 rs1800872* gene polymorphism and the SCC risk were significantly associated in both models (CC/AA: OR = 0.59, 95% CI: 0.44–0.81, *p* = 0.001; CC/AA + AC: OR = 0.71, 95% CI: 0.59–0.86, *p* = 0.000; Figure 3). However, the *IL-10 rs1800872* gene polymorphism and the SCC risk showed no significant association in the other two models (AC/AA: OR = 0.87, 95% CI: 0.69–1.11, *p* = 0.266; AC + CC/AA: OR = 0.78, 95% CI: 0.60–1.00, *p* = 0.050; Figure 3). The subgroup analysis by ethnicity revealed a significantly increased SCC risk in Caucasians in all models (CC/AA: OR = 0.48, 95% CI: 0.31–0.74, *p* = 0.001; AC/AA: OR = 0.68, 95% CI: 0.54–0.86, *p* = 0.001; AC + CC/A: OR = 0.58, 95% CI: 0.46–0.72, *p* = 0.000; CC/AA + AC: OR = 0.68, 95% CI: 0.52–0.89, *p* = 0.004). Similarly, Asians showed an increased risk (CC/AA + AC: OR = 0.80, 95% CI: 0.63–1.00, *p* = 0.049). The subgroup analysis by control source revealed a significantly increased SCC risk among population-based controls in both models (CC/AA: OR = 0.55, 95% CI: 0.41–0.73, *p* = 0.000; CC/AA + AC: OR = 0.73, 95% CI: 0.61–0.87, *p* = 0.000) and a significantly increased risk among hospital-based controls in only one model (CC/AA + AC: OR = 0.69, 95% CI: 0.48–0.98, *p* = 0.036). The subgroup analysis by cancer type revealed a significantly increased oral SCC risk (CC/AA: OR = 0.28, 95% CI: 0.17–0.49, *p* = 0.000; AC/AA: OR = 0.39, 95% CI: 0.24–0.62, *p* = 0.000; AC + CC/AA: OR = 0.36, 95% CI: 0.23–0.57, *p* = 0.000; CC/AA + AC: OR = 0.63, 95% CI: 0.44–0.89, *p* = 0.000). The risk of cervical SCC was also significantly increased (CC/AA: OR = 0.46, 95% CI: 0.25–0.84, *p* = 0.011; AC + CC/AA: OR = 0.62, 95% CI: 0.46–0.83, *p* = 0.001; CC/AA + AC: OR = 0.54, 95% CI: 0.35–0.83, *p* = 0.005). All gene models showed heterogeneity (CC/AA: *I* ^2^ = 61.40%, *p* = 0.002; AC/AA: *I* ^2^ = 65.10%, *p* = 0.001; AC + CC/AA: *I* ^2^ = 72.20%, *p* = 0.000; CC/AA + AC: *I* ^2^ = 51.80%, *p* = 0.023).

**Table 5:** Stratified analyses of the *IL-10 rs1800872* polymorphism on squamous cell carcinoma risk

Comparative model	No.	*Z*	*p*	OR (95% CI)	Heterogeneity			*Z*	Begg's test	*t*	Egger's test	FPRP *p*-value	FPRP Statistical power	FPRP prior probability					BEDP prior probability		
					Heterogeneity chi-squared	*p*	*I* ^2^							0.250	0.10	0.01	0.001	0.0001	0.01	0.001	0.000001
**CC/AA**																					
Overall	11	3.34	0.001	0.595(0.438–0.807)	27.83	0.002	61.40%	0.93	0.350	−0.83	0.428	**0.001**	0.232	**0.011**	0.032	0.264	0.783	0.973	**0.677**	0.955	1.000
**Ethnicity**																					
Caucasian	7	3.35	0.001	0.481(0.314–0.738)	16.55	0.011	63.80%	0.00	1.000	0.14	0.895	**0.001**	**0.068**	**0.035**	**0.097**	0.541	0.923	0.992	**0.676**	0.955	1.000
Asian	4	1.77	0.076	0.807(0.636–1.023)	2.57	0.462	0.00%	−0.34	1.000	0.01	0.991	**0.076**	0.943	**0.196**	0.422	0.889	0.988	0.999	0.991	0.999	1.000
**Source of control**																					
HB	5	1.93	0.054	0.635(0.401–1.007)	16.71	0.002	76.10%	0.73	0.462	−0.73	0.518	**0.054**	0.418	0.278	0.536	0.927	0.992	0.999	0.981	0.998	1.000
PB	6	4.12	0.000	0.550(0.413–0.730)	9.85	0.080	49.20%	0.38	0.707	0.14	0.895	**0.000**	**0.091**	**0.001**	**0.003**	**0.036**	0.276	0.792	**0.114**	**0.566**	0.992
**Cancer types**																					
Laryngeal SCC	2	0.67	0.505	0.852(0.533–1.363)	0.01	0.924	0.00%	0.00	1.000	–	–	0.504	0.847	0.641	0.843	0.983	0.998	1.000	0.995	1.000	1.000
Oral SCC	2	4.61	0.000	0.284(0.167–0.485)	0.38	0.538	0.00%	0.00	1.000	–	–	**0.000**	**0.001**	**0.013**	**0.039**	0.310	0.819	0.978	**0.040**	**0.298**	0.977
Cervical SCC	3	2.54	0.011	0.458(0.250–0.836)	6.60	0.037	69.70%	0.00	1.000	−0.15	0.906	**0.011**	**0.111**	0.229	0.472	0.908	0.990	0.999	0.945	0.994	1.000
Esophageal SCC	4	1.63	0.102	0.817(0.640–1.041)	2.90	0.408	0.00%	−0.34	1.000	0.40	0.727	**0.102**	0.950	0.244	0.492	0.914	0.991	0.999	0.992	0.999	1.000
**Genotyping method**																					
PCR	7	2.60	0.009	0.613(0.423–0.887)	19.63	0.003	69.40%	1.50	0.133	−1.19	0.287	**0.009**	0.328	0.079	0.206	0.740	0.966	0.997	0.938	0.993	1.000
PCR-RFLP	3	2.1	0.036	0.490(0.252–0.953)	5.54	0.063	63.90%	0.00	1.000	−0.07	0.953	**0.035**	**0.182**	0.369	0.637	0.951	0.995	0.999	0.974	0.997	1.000
**AC/AA**																					
Overall	11	1.11	0.266	0.872(0.686–1.110)	28.64	0.001	65.10%	0.93	0.350	−0.84	0.423	0.266	0.985	0.447	0.708	0.964	0.996	1.000	0.996	1.000	1.000
**Ethnicity**																					
Caucasian	7	3.24	0.001	0.679(0.537–0.858)	9.85	0.131	39.10%	0.60	0.548	−0.16	0.880	**0.001**	0.561	**0.006**	**0.019**	**0.173**	0.678	0.955	**0.757**	0.969	1.000
Asian	4	0.77	0.438	1.126(0.835–1.518)	10.38	0.016	71.10%	1.02	0.308	1.37	0.304	0.436	0.970	0.574	0.802	0.978	0.998	1.000	0.996	1.000	1.000
**Source of control**																					
HB	5	0.43	0.670	0.946(0.734–1.220)	8.51	0.075	53.00%	−0.24	1.000	−0.37	0.736	0.669	0.996	0.668	0.858	0.985	0.999	1.000	0.998	1.000	1.000
PB	6	0.94	0.349	0.792(0.487–1.289)	19.28	0.002	74.10%	0.38	0.707	−0.47	0.663	0.348	0.756	0.580	0.806	0.979	0.998	1.000	0.994	0.999	1.000
**Cancer types**																					
Laryngeal SCC	2	0.73	0.463	1.298(0.647–2.603)	3.07	0.080	67.40%	0.00	1.000	–	–	0.463	0.658	0.678	0.863	0.986	0.999	1.000	0.993	0.999	1.000
Oral SCC	2	3.95	0.000	0.387(0.241–0.620)	0.24	0.624	0.00%	0.00	1.000	–	–	**0.000**	**0.012**	**0.020**	**0.056**	0.397	0.869	0.985	**0.258**	**0.778**	0.997
Cervical SCC	3	1.32	0.188	0.808(0.588–1.110)	2.10	0.350	4.60%	1.04	0.296	1.57	0.361	**0.188**	0.882	0.390	0.658	0.955	0.995	1.000	0.994	0.999	1.000
Esophageal SCC	4	0.15	0.878	0.988(0.852–1.146)	5.54	0.136	45.80%	0.34	0.734	0.21	0.850	0.873	1.000	0.724	0.887	0.989	0.999	1.000	0.999	1.000	1.000
**Genotyping method**																					
PCR	7	0.94	0.347	0.889(0.695–1.137)	13.24	0.039	54.70%	0.60	0.548	−0.92	0.402	0.348	0.989	0.514	0.760	0.972	0.997	1.000	0.997	1.000	1.000
PCR-RFLP	3	0.44	0.658	0.835(0.376–1.853)	15.33	0.000	86.90%	0.00	1.000	−0.26	0.835	0.657	0.710	0.735	0.893	0.989	0.999	1.000	0.994	0.999	1.000
**AC+CC/AA**																					
Overall	11	1.96	0.050	0.775(0.601–1.000)	35.98	0.000	72.20%	1.09	0.276	−1.17	0.272	**0.050**	0.877	**0.146**	0.339	0.850	0.983	0.998	0.986	0.999	1.000
**Ethnicity**																					
Caucasian	7	4.83	0.000	0.579(0.463–0.722)	10.94	0.090	45.20%	0.30	0.764	0.29	0.784	**0.000**	**0.105**	**0.000**	**0.000**	**0.001**	**0.011**	**0.104**	**0.006**	**0.058**	0.861
Asian	4	0.36	0.717	1.052(0.799–1.384)	9.77	0.021	69.30%	0.34	0.734	1.02	0.415	0.717	0.994	0.684	0.867	0.986	0.999	1.000	0.997	1.000	1.000
**Source of control**																					
HB	5	1.17	0.241	0.822(0.592–1.141)	15.91	0.003	74.90%	−0.24	1.000	−0.78	0.495	0.241	0.895	0.447	0.708	0.964	0.996	1.000	0.995	0.999	1.000
PB	6	1.38	0.167	0.724(0.457–1.144)	18.78	0.002	73.40%	0.00	1.000	−0.46	0.669	**0.166**	0.638	0.439	0.701	0.963	0.996	1.000	0.991	0.999	1.000
**Cancer types**																					
Laryngeal SCC	2	0.60	0.548	1.182(0.685–2.041)	2.15	0.143	53.50%	0.00	1.000	–	–	0.549	0.804	0.972	0.860	0.985	0.999	1.000	0.995	0.999	1.000
Oral SCC	2	4.37	0.000	0.358(0.226–0.567)	0.27	0.606	0.00%	0.00	1.000	–	–	**0.000**	**0.004**	**0.009**	**0.026**	0.227	0.748	0.967	**0.069**	**0.426**	0.987
Cervical SCC	3	3.20	0.001	0.615(0.457–0.829)	3.44	0.179	41.80%	1.04	0.296	1.59	0.357	**0.001**	0.298	**0.014**	**0.041**	0.320	0.826	0.979	0.767	0.971	1.000
Esophageal SCC	4	0.20	0.843	0.975(0.762–1.249)	6.44	0.092	53.40%	0.34	0.734	0.30	0.793	0.841	0.999	0.716	0.883	0.988	0.999	1.000	0.998	1.000	1.000
**Genotyping method**																					
PCR	7	1.78	0.076	0.767(0.573–1.028)	21.01	0.002	71.40%	0.60	0.548	−1.37	0.229	0.075	0.826	0.216	0.453	0.901	0.989	0.999	0.989	0.999	1.000
PCR-RFLP	3	0.75	0.451	0.751(0.357–1.581)	14.70	0.001	86.40%	0.00	1.000	−0.36	0.778	0.451	0.623	0.685	0.867	0.986	0.999	1.000	0.993	0.999	1.000
**CC/AA+AC**																					
Overall	11	3.59	0.000	0.711(0.590–0.857)	20.75	0.023	51.80%	0.00	1.000	−0.72	0.487	**0.000**	0.750	**0.001**	**0.004**	**0.043**	0.314	0.821	**0.539**	0.922	0.999
**Ethnicity**																					
Caucasian	7	2.85	0.004	0.677(0.518–0.885)	18.33	0.005	67.30%	0.00	1.000	−0.64	0.553	**0.004**	0.545	**0.023**	**0.067**	0.440	0.888	0.988	0.900	0.989	1.000
Asian	4	1.97	0.049	0.796(0.634–0.999)	1.66	0.647	0.00%	0.34	0.734	−0.87	0.474	**0.049**	0.937	**0.136**	0.320	0.838	0.981	0.998	0.987	0.999	1.000
**Source of control**																					
HB	5	2.09	0.036	0.687(0.484–0.976)	16.55	0.002	75.80%	0.24	0.806	−1.15	0.332	**0.036**	0.567	0.161	0.365	0.863	0.985	0.998	0.978	0.998	1.000
PB	6	3.54	0.000	0.729(0.612–0.868)	4.19	0.522	0.00%	0.75	0.452	−0.05	0.961	**0.000**	0.842	**0.001**	**0.004**	**0.043**	0.314	0.821	**0.575**	0.932	0.999
**Cancer types**																					
Laryngeal SCC	2	0.73	0.463	0.904(0.690–1.184)	0.95	0.329	0.00%	0.00	1.000	–	–	0.463	0.987	0.585	0.809	0.979	0.998	1.000	0.997	1.000	1.000
Oral SCC	2	2.62	0.000	0.625(0.440–0.888)	0.01	0.920	0.00%	0.00	1.000	–	–	**0.009**	0.359	**0.068**	**0.179**	0.706	0.960	0.996	0.935	0.993	1.000
Cervical SCC	3	2.79	0.005	0.538(0.348–0.831)	8.23	0.016	75.70%	0.00	1.000	−3.11	0.198	**0.005**	**0.167**	**0.085**	0.219	0.755	0.969	0.997	0.902	0.989	1.000
Esophageal SCC	4	1.34	0.181	0.860(0.689–1.073)	2.91	0.406	0.00%	0.34	0.734	0.48	0.678	**0.182**	0.988	0.355	0.623	0.948	0.995	0.999	0.995	1.000	1.000
**Genotyping method**																					
PCR	7	2.93	0.003	0.700(0.551–0.888)	16.81	0.01	64.30%	0.30	0.764	−1.10	0.321	**0.003**	0.656	**0.015**	**0.043**	0.332	0.834	0.980	0.883	0.987	1.000
PCR-RFLP	3	2.98	0.003	0.625(0.459–0.852)	0.01	0.996	0.00%	0.00	1.000	0.67	0.626	**0.003**	0.342	**0.025**	**0.072**	0.461	0.896	0.989	0.859	0.984	1.000

Abbreviations: OR, odds ratio; CI, confidence interval; PB, population-based; HB, hospital-based; SCC, squamous cell carcinoma; FPRP, false positive report probability; BFDP, Bayesian false discovery probability. The results in bold represented that there was statistically significant noteworthiness at 0.2 level by FPRP or 0.8 level by BFDP calculations.

**Figure 4:**
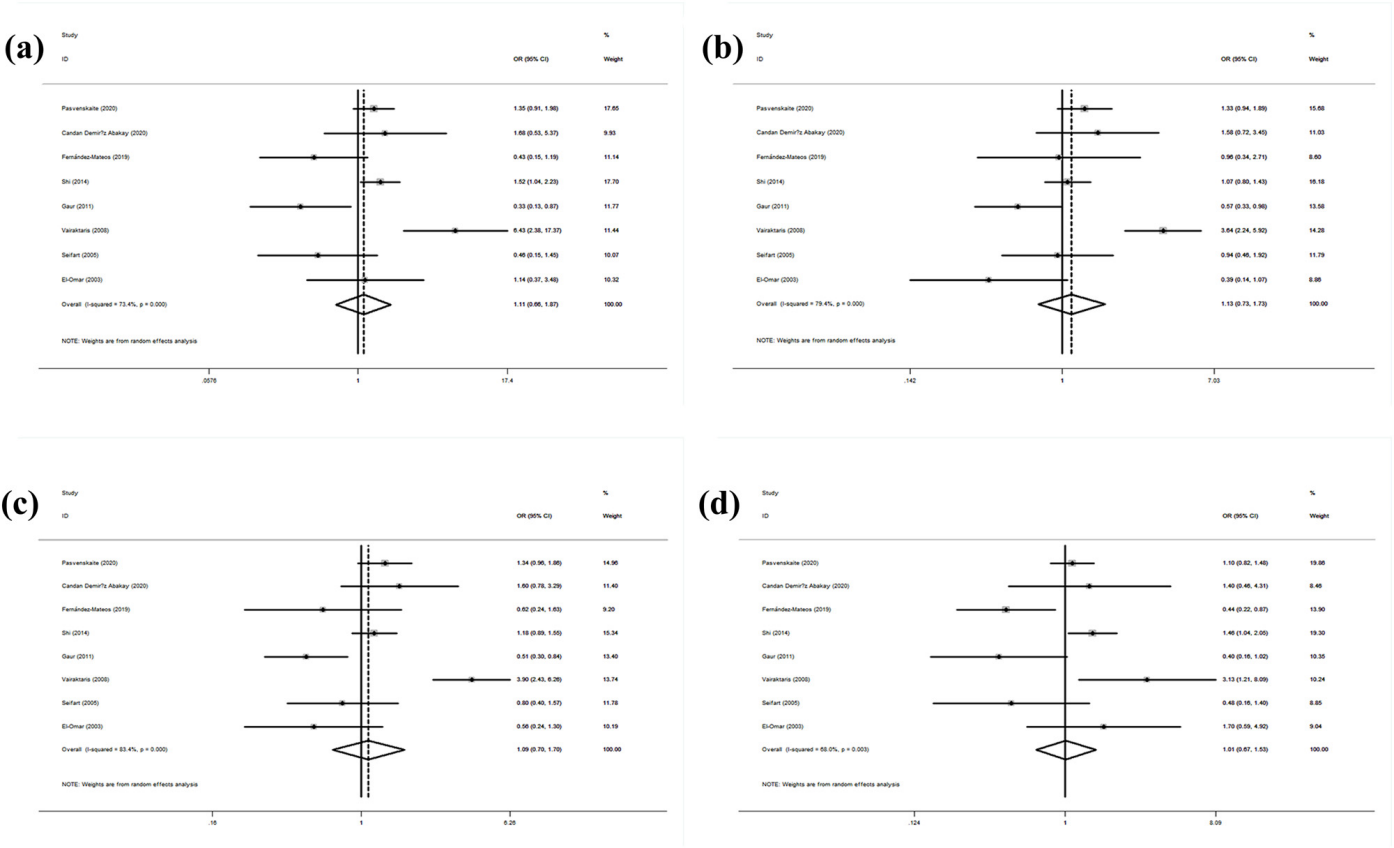
Statistical relationship between *IL-10 rs1800795* gene polymorphism and squamous cell carcinoma susceptibility in four models: (a) CC vs GG, (b) GC vs GG, (c) GC + CC vs GG, and (d) CC vs GG + GC.

Table 6 shows the association between the *IL-6 rs1800795* gene polymorphism and the SCC risk. The association between the *IL-6 rs1800795* gene polymorphism and the SCC risk was explored in all models (CC/GG: OR = 1.11, 95% CI: 0.66–1.87, *p* = 0.702; GC/GG: OR = 1.13, 95% CI: 0.74–1.73, *p* = 0.58; GC + CC/GG: OR = 1.09, 95% CI: 0.70–1.70, *p* = 0.697; CC/GG + GC: OR = 1.01, 95% CI: 0.67–1.53, *p* = 0.958; Figure 4). The subgroup analysis by ethnicity showed no significant association in any model. The subgroup analysis by control source revealed a significantly increased risk among hospital-based controls in both models (CC/GG: OR = 1.43, 95% CI: 1.09–1.88, *p* = 0.009; GC + CC/GG: OR = 1.24, 95% CI: 1.01–1.53, *p* = 0.044). The subgroup analysis by cancer type revealed a significantly increased laryngeal SCC risk in one model (GC + CC/GG: OR = 1.38, 95% CI: 1.02–1.86, *p* = 0.035). All gene models showed heterogeneity (CC/GG: *I* ^2^ = 73.40%, *p* = 0.000; GC/GG: *I* ^2^ = 79.40%, *p* = 0.000; GC + CC/GG: *I* ^2^ = 83.40%, *p* = 0.000; CC/GG + GC: *I* ^2^ = 68.00%, *p* = 0.003). No other results were statistically significant.

**Table 6:** Stratified analyses of the *IL-6 rs1800795* polymorphism on squamous cell carcinoma risk

Comparative model	No.	*Z*	*p*	OR (95% CI)	Heterogeneity			*Z*	Begg’s test	*t*	Egger’s test	FPRP *p*-value	FPRP statistical power	FPRP prior probability					BEDP prior probability		
					Heterogeneity chi-squared	*p*	*I* ^2^							0.25	0.10	0.01	0.001	0.0001	0.01	0.001	0.000001
**CC/GG**																					
Overall	8	0.38	0.702	1.107(0.657–1.867)	26.31	0.000	73.40%	0.12	0.902	−0.75	0.479	0.703	0.873	0.707	0.879	0.988	0.999	1.000	0.995	1.000	1.000
**Ethnicity**																					
Caucasian	6	0.61	0.54	1.244(0.619–2.497)	17.88	0.003	72.00%	0.00	1.000	−0.2	0.849	0.539	0.701	0.698	0.874	0.987	0.999	1.000	0.994	0.999	1.000
Asian	2	0.36	0.717	0.759(0.171–3.368)	8.39	0.004	88.10%	0.00	1.000	–	–	0.717	0.568	0.791	0.919	0.992	0.999	1.000	0.992	0.999	1.000
**Source of control**																					
HB	2	2.6	0.009	1.433(1.093–1.878)	0.20	0.654	0.00%	0.00	1.000	–	–	**0.009**	0.630	**0.042**	**0.115**	0.589	0.935	0.993	0.944	0.994	1.000
PB	6	0.08	0.938	0.963(0.374–2.479)	23.75	0.000	78.90%	0.75	0.452	0.07	0.944	0.938	0.777	0.784	0.916	0.992	0.999	1.000	0.993	0.999	1.000
**Cancer types**																					
Oral SCC	3	0.03	0.974	0.969(0.150–6.274)	21.24	0.000	90.60%	0.00	1.000	0.13	0.916	0.974	0.653	0.817	0.931	0.993	0.999	1.000	0.991	0.999	1.000
Laryngeal SCC	2	1.71	0.087	1.377(0.955–1.985)	0.13	0.723	0.00%	0.00	1.000	–	–	**0.086**	0.677	0.277	0.535	0.927	0.992	0.999	0.988	0.999	1.000
**Genotyping method**																					
PCR	5	0.54	0.587	1.258(0.549–2.884)	17.81	0.001	77.50%	−0.24	1.000	−0.15	0.892	**0.587**	0.661	0.727	0.889	0.989	0.999	1.000	0.993	0.999	1.000
PCR-RFLP	2	0.36	0.717	0.759(0.171–3.368)	8.39	0.004	88.10%	0.00	1.000	–	–	0.72	0.568	0.791	0.919	**0.992**	0.999	1.000	0.988	0.999	1.000
**GC/GG**																					
Overall	8	0.55	0.584	1.127(0.735–1.730)	34.02	0.000	79.40%	−0.12	1.000	−0.48	0.647	0.585	0.904	0.660	0.853	0.985	0.998	1.000	0.996	1.000	1.000
**Ethnicity**																					
Caucasian	6	0.87	0.386	1.282(0.731–2.250)	22.44	0.000	77.70%	0.38	0.707	−0.93	0.404	0.387	0.708	0.621	0.831	0.982	0.998	1.000	0.994	0.999	1.000
Asian	2	0.65	0.516	0.814(0.438–1.514)	4.06	0.044	75.30%	0.00	1.000	–	–	0.516	0.736	0.678	0.863	0.986	0.999	1.000	0.994	0.999	1.000
**Source of control**																					
HB	2	1.41	0.158	1.174(0.940–1.467)	0.89	0.345	0.00%	0.00	1.000	–	–	**0.158**	0.984	0.325	0.591	0.941	0.994	0.999	0.995	0.999	1.000
PB	6	0.15	0.880	1.059(0.503–2.230)	32.88	0.00	84.80%	0.00	1.000	−1.14	0.319	0.880	0.820	0.763	0.906	0.990	0.999	1.000	0.997	1.000	1.000
**Cancer types**																					
Oral SCC	3	0.36	0.718	1.276(0.339–4.807)	25.62	0.00	92.20%	0.00	1.000	−0.38	0.769	0.719	0.594	0.784	0.916	0.992	0.999	1.000	0.992	0.999	1.000
Laryngeal SCC	2	1.95	0.051	1.371(0.998–1.884)	0.15	0.702	0.00%	0.00	1.000	–	–	**0.052**	0.710	**0.179**	0.396	0.878	0.986	0.999	0.984	0.998	1.000
**Genotyping method**																					
PCR	5	1.63	0.103	1.554(0.915–2.640)	15.12	0.004	73.50%	0.24	0.806	−0.33	0.763	**0.103**	0.448	0.408	0.674	0.958	0.996	1.000	0.987	0.999	1.000
PCR-RFLP	2	0.65	0.516	0.814(0.438–1.514)	4.06	0.044	75.30%	0.00	1.000	–	–	0.515	0.736	0.678	0.863	0.986	0.999	1.000	0.994	0.999	1.000
**GC + CC/GG**																					
Overall	8	0.39	0.697	1.092(0.701–1.700)	42.10	0.000	83.40%	0.62	0.536	−0.66	0.536	0.697	0.920	0.694	0.872	0.989	0.999	1.000	0.996	1.000	1.000
**Ethnicity**																					
Caucasian	6	0.68	0.494	1.225(0.685–2.191)	27.83	0.000	82.00%	0.75	0.452	−0.96	0.393	0.494	0.753	0.663	0.855	0.985	0.998	1.000	0.994	0.999	1.000
Asian	2	0.55	0.584	1.092(0.701–1.700)	8.15	0.004	87.70%	0.00	1.000	–	–	0.697	0.920	0.694	0.872	0.987	0.999	1.000	0.996	1.000	1.000
**Source of control**																					
HB	2	2.01	0.044	1.241(1.006–1.532)	0.35	0.556	0.00%	0.00	1.000	–	–	**0.045**	0.961	**0.122**	0.294	0.821	0.979	0.998	0.987	0.999	1.000
PB	6	0.00	0.997	1.002(0.461–2.178)	41.69	0.000	88.00%	0.38	0.707	−0.99	0.380	0.996	0.846	0.779	0.914	0.991	0.999	1.000	0.994	0.999	1.000
**Cancer types**																					
Oral SCC	3	0.12	0.906	1.093(0.250–4.792)	35.92	0.000	94.40%	0.00	1.000	−0.42	0.747	0.906	0.663	0.804	0.925	0.993	0.999	1.000	0.992	0.999	1.000
Laryngeal SCC	2	2.11	0.035	1.380(1.024–1.860)	0.2	0.655	0.00%	0.00	1.000	–	–	**0.034**	0.708	**0.127**	0.304	0.828	0.980	0.998	0.979	0.998	1.000
**Genotyping method**																					
PCR	5	1.10	0.269	1.408(0.767–2.586)	22.43	0.000	82.20%	0.24	0.806	−0.52	0.639	0.269	0.581	0.582	0.807	0.979	0.998	1.000	0.992	0.999	1.000
PCR-RFLP	2	0.55	0.584	0.794(0.348–1.811)	8.15	0.004	87.70%	0.00	1.000	–	–	0.583	0.661	0.726	0.888	0.989	0.999	1.000	0.993	0.999	1.000
**CC/GG + GC**																					
Overall	8	0.05	0.958	1.011(0.667–1.532)	21.84	0.003	68.00%	0.12	0.902	−0.60	0.570	0.959	0.969	0.748	0.899	0.990	0.999	1.000	0.996	1.000	1.000
**Ethnicity**																					
Caucasian	6	0.19	0.846	1.055(0.614–1.811)	14.49	0.013	65.50%	0.00	1.000	0.07	0.945	0.846	0.899	0.738	0.894	0.989	0.999	1.000	0.995	1.000	1.000
Asian	2	0.30	0.765	0.825(0.233–2.921)	6.48	0.011	84.60%	0.00	1.000	–	–	0.766	0.629	0.785	0.916	0.992	0.999	1.000	0.992	0.999	1.000
**Source of control**																					
HB	2	1.94	0.053	1.247(0.997–1.559)	1.51	0.219	33.70%	0.00	1.000	–	–	**0.053**	0.948	**0.143**	0.334	0.846	0.982	0.998	0.988	0.999	1.000
PB	6	0.29	0.769	0.899(0.441–1.832)	16.70	0.005	70.10%	0.75	0.452	1.12	0.327	0.769	0.795	0.744	0.897	0.990	0.999	1.000	0.994	0.999	1.000
**Cancer types**																					
Oral SCC	3	0.35	0.728	0.802(0.232–2.774)	12.71	0.002	84.30%	1.04	0.296	0.66	0.629	0.727	0.615	0.780	0.914	0.992	0.999	1.000	0.992	0.999	1.000
Laryngeal SCC	2	0.77	0.441	1.120(0.839–1.494)	0.17	0.683	0.00%	0.00	1.000	–	–	0.441	0.977	0.575	0.802	0.978	0.998	1.000	0.997	1.000	1.000
**Genotyping method**																					
PCR	5	0.06	0.951	0.981(0.531–1.813)	13.62	0.009	70.60%	−0.24	1.000	−0.13	0.9204	0.951	0.891	0.762	0.906	0.991	0.999	1.000	0.995	1.000	1.000
PCR-RFLP	2	0.3	0.765	0.825(0.233–2.921)	6.48	0.011	84.60%	0.00	1.000	–	–	0.765	0.629	0.785	0.916	0.992	0.999	1.000	0.992	0.999	1.000

Abbreviations: OR, odds ratio; CI, confidence interval; PB, population-based; HB, hospital-based; SCC, squamous cell carcinoma; FPRP, false positive report probability; BFDP, Bayesian false discovery probability. The results in bold represented that there was statistically significant noteworthiness at 0.2 level by FPRP or 0.8 level by BFDP calculations.

### 3.3 Publication bias

The funnel plot revealed no significant asymmetry in the *IL-10 rs1800896* gene polymorphism in any model (Figure S1). However, Egger’s test revealed a publication bias in the *IL-10 rs1800896* models (*p* = 0.018 for GG/AA; *p* = 0.037 for GG/AA + AG). *IL-18 rs1800872* or *IL-6 rs1800795* models showed no asymmetry in the funnel shape (Figures S2 and S3). In addition, Egger’s test revealed no publication bias in *IL-10 1800872* or *IL-6 rs1800795* models.

### 3.4 Sensitivity analysis

The sensitivity analysis revealed no significant changes in the combination OR corresponding to the *IL-10 rs1800896* or *rs1800872* or *IL-6 rs1800795* gene polymorphism, proving our results to be statistically robust (Figures S4–S6). The meta-regression analysis showed that the publication year, ethnicity, or control source did not affect the stability of the combined results (Table S1).

### 3.5 FPRP and BFDP tests

Tables S2, S3, and S4 show the FPRPs of *IL-10 rs1800896* and *rs1800872* and *IL-6 rs1800795* gene polymorphisms, respectively. At prior probabilities of 0.25 and 0.1, FPRP and BFDP test results showed statistically significant results in almost all models of *rs1800896*, rs1800872, and rs1800795 polymorphisms, with an OR of 1.5.

## 4 Discussion

SCC can be caused by exposure to carcinogens, such as sunlight, tobacco, alcohol, and viral infections. It shows a high percentage of somatic genetic mutations. All SCC cases have similar mutation patterns [44–46]. The relationship between the *IL-10* and *IL-6* gene polymorphisms and the SCC risk has been shown. Gene polymorphisms affect their messenger RNA (mRNA) and protein levels. The *IL-6 rs1800795* polymorphism may affect the *IL-6* mRNA expression. The G allele of the *IL-6 rs1800795* promoter single-nucleotide polymorphism is associated with elevated *IL-6* mRNA transcription levels after *in vitro* endotoxin or *IL-1* stimulation [47]. The presence of a variant allele G in tumor tissue is positively associated with elevated *IL-10* mRNA levels [48]. Wang et al. analyzed the same polymorphisms and reported significantly higher *IL-10* mRNA levels in patients with non-small cell lung cancer with the non-ATA haplotype, showing the association of cytokine *IL-10* expression levels with tumor progression [49]. Currently, no relevant reports have been published on the polymorphism of these three genes or proteins. They may indirectly affect the protein expression after affecting the mRNA expression. Associations of *IL-10* and *IL-6* gene polymorphisms and oral and cervical SCC risks have been frequently demonstrated in meta-analyses [50,51]. However, no meta-analysis has revealed an association of *IL-10* or *IL-6* gene polymorphisms with the SCC risk. Therefore, we re-examined the relationship between *IL-10* and *IL-6* gene polymorphisms and the SCC risk from a comprehensive and unified perspective to draw a more accurate conclusion.

We investigated the *IL-10 rs1800896* gene polymorphism to find its relationship with the SCC risk. The overall analysis revealed no positive results. However, the subgroup analysis by cancer type revealed positive results, indicating that the *IL-10 rs1800896* gene polymorphism was a risk factor for oral SCC. Li et al. [50] also conducted a study on the association of the *IL-10* gene polymorphism and the oral cancer risk. They reported that the *IL-10 rs1800896* polymorphism increased the risk of oral cancers, including non-SCC, in both dominant and recessive genetic models. Both present and previous studies showed that the *IL-10 rs1800896* gene polymorphism increased the oral cancer risk. However, the present study investigated SCC, while previous studies did not differentiate SCC and non-SCC. In addition, the present subgroup analysis showed that other cancer types, such as cervical or esophageal SCC, showed no positive results. These results were consistent with those of Ni et al.’s meta-analysis of eight studies. Furthermore, the *IL-10 rs1800896* gene polymorphism affects carcinoma of the uterine cervix [52]. However, the present results were inconsistent with the results of Li et al.’s meta-analysis of seven studies, which showed that the *IL-10 rs1800896* gene polymorphism could increase the risk of esophageal cancer, possibly including non-SCC [53]. In the present study, the *IL-10 rs1800896* polymorphism showed different impressions in diverse organs, probably because of the *IL-10 rs1800896* gene polymorphisms in different parts of the body having different distributions in different cancer types. However, the exact mechanism remains unclear. The subgroup analyses did not include a sufficient sample size. Therefore, we should exercise caution in drawing conclusions. Future, large-scale studies should be conducted. No link between the *IL-10 rs1800896* gene polymorphism and the SCC risk was found in hospital- or population-based models.

With the *IL-10 rs1800872* gene polymorphism, the SCC risk was low, particularly in oral SCC. Subgroup analyses by ethnicity indicated that the *IL-10 rs1800872* polymorphism might be a protective factor in the Caucasian population but a risk factor in the Asian population. The *IL-10 rs1800872* gene polymorphism had different effects on the Caucasian and Asian populations. The reason might be that the proportion of the gene expression differed among ethnic groups. These differences may be derived from different genetic backgrounds and environmental exposures, such as the difference in minor allele frequencies in healthy controls among the Caucasian and Asian populations. Therefore, inconsistent associations indicate the possibility of differences in the magnitude of the *IL-10 rs1800872* gene polymorphism contribution to the SCC risk across different genetic backgrounds and environmental exposures [54]. The subgroup analysis by cancer type showed no association between the *IL-10 rs1800872* polymorphism and the esophageal SCC risk, broadly consistent with the included independent studies [20,24,36,37].

The *IL-6 rs1800795* gene polymorphism and the SCC risk showed no association. However, the subgroup analysis by control source revealed that the presence of the *IL-6 rs1800795* gene polymorphism increased the SCC risk among hospital-based controls but not among population-based controls. The possible reasons are as follows: (1) some studies included hospital-based controls, which could induce an inherent selection bias because the hospital population does not represent the general population and (2) hospital-based controls may have other diseases that affect the release of interleukins, affecting the present results. Thus, appropriate and representative control populations played roles in assessing the relationship between the gene polymorphism and the disease risk. We found inconsistent results for the same cancer among independent studies. One study showed that the CC genotype of *IL-6 rs1800795* may be protective in patients with oral SCC [42]. Another study showed a seven-fold increased risk of oral SCC with the CC genotype [43]. The results of the two studies were directly opposite [42,43]; therefore, we performed a subgroup analysis by cancer type. However, the results showed no significant association between the *IL-6 rs1800795* polymorphism and the oral SCC risk, consistent with previous meta-analysis results [55]. A previous meta-analysis of 11 studies conducted by Rezaei et al. also confirmed that the *IL-6 rs1800795* gene polymorphism was not associated with the oral cancer risk. Thus, the strength of the association between the *IL-6 rs1800795* gene polymorphism and the oral cancer risk could be evaluated using meta-analyses, and the results of this study were more accurate than those of each independent study. All studies that met the eligibility criteria were included in this study, but a larger sample size would increase the reliability of the conclusions.

This meta-analysis has some advantages. First, no studies on the association between the *IL-10 rs1800896* and *rs1800872* and *IL-6 rs1800795* gene polymorphisms and the SCC risk have been reported. Second, this was the most comprehensive study on this topic, with sufficient statistical power. However, several limitations exist. First, many studies had to be excluded because they did not report the relevant cancer type or pathologically diagnosed SCC. Further, some studies had to be excluded because they did not report the proportion of SCC by genotype. Finally, the number of included studies was small. Future, large-scale studies are required to more accurately explain the association between the studied genotypes and the SCC risk. Second, we only included articles published in English or Chinese, which might lead to a language bias. The preponderance of Asians in the original data could also be a bias. Third, the pathogenesis of SCC was affected by various factors, such as environmental changes, diet, age, and sex, which were not accounted for because of the retrospective study design. Fourth, subgroup analyses included only a few studies, reducing the statistical efficiency. Finally, the statistical heterogeneity frequently existed between the *IL-10 rs1800872* and *IL-6 rs1800795* gene polymorphisms and the SCC risk during statistical calculations. This could be because of the small number of included studies and the considerable heterogeneity among studies. Therefore, caution should be exercised when drawing conclusions.

## 5 Conclusions

This meta-analysis showed that the *IL-10 rs1800872* gene polymorphism reduced the SCC risk, particularly in Caucasians. However, no *IL-10 rs1800896* or *IL-6 rs1800795* polymorphism was correlated with the SCC risk. Considering the limitations of this study, further carefully designed, large-scale studies are required to evaluate the association of the *IL-10* and *IL-6* genetic polymorphisms with the SCC risk.

## Supplementary Material

Supplementary material
