# Supplementary material for: Association of interleukin-10 rs1800896, rs1800872, and interleukin-6 rs1800795 polymorphisms with squamous cell carcinoma risk: A meta-analysis
Source: Open Life Sci. 2023 Apr 15;18(1):20220580. doi: 10.1515/biol-2022-0580 (PMC10106975; doi:10.1515/biol-2022-0580)
Supplement: Supplementary material [file biol-2022-0580-sm.pdf]

Supplementary material

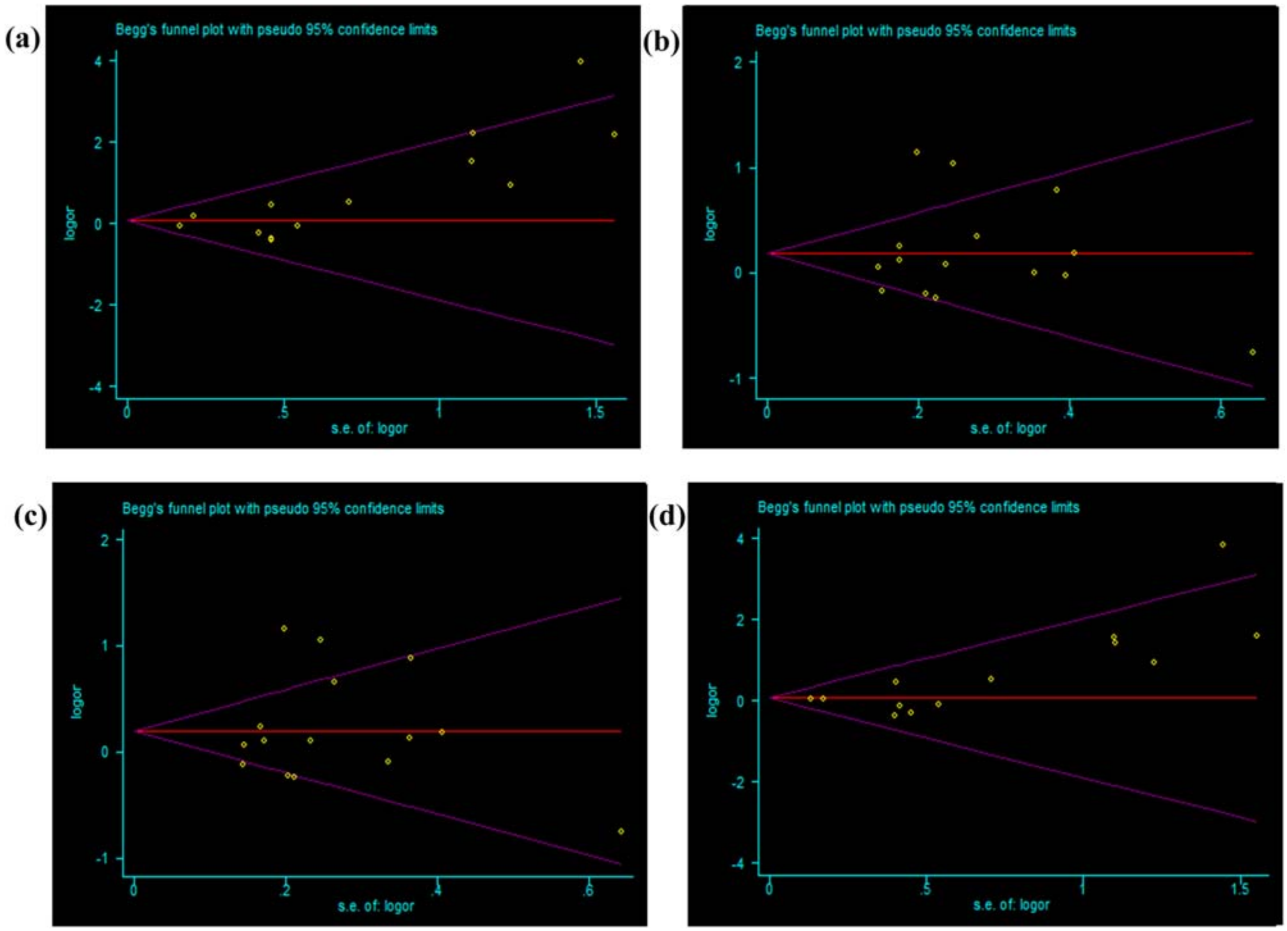

Figure S1: Begg's funnel plots for the relationship between the *IL-10 rs1800896* gene polymorphism and the squamous cell carcinoma risk in four models. A: GG vs. AA; B: AG vs. AA; C: AG+GG vs. AA; D: GG vs. AA+AG.

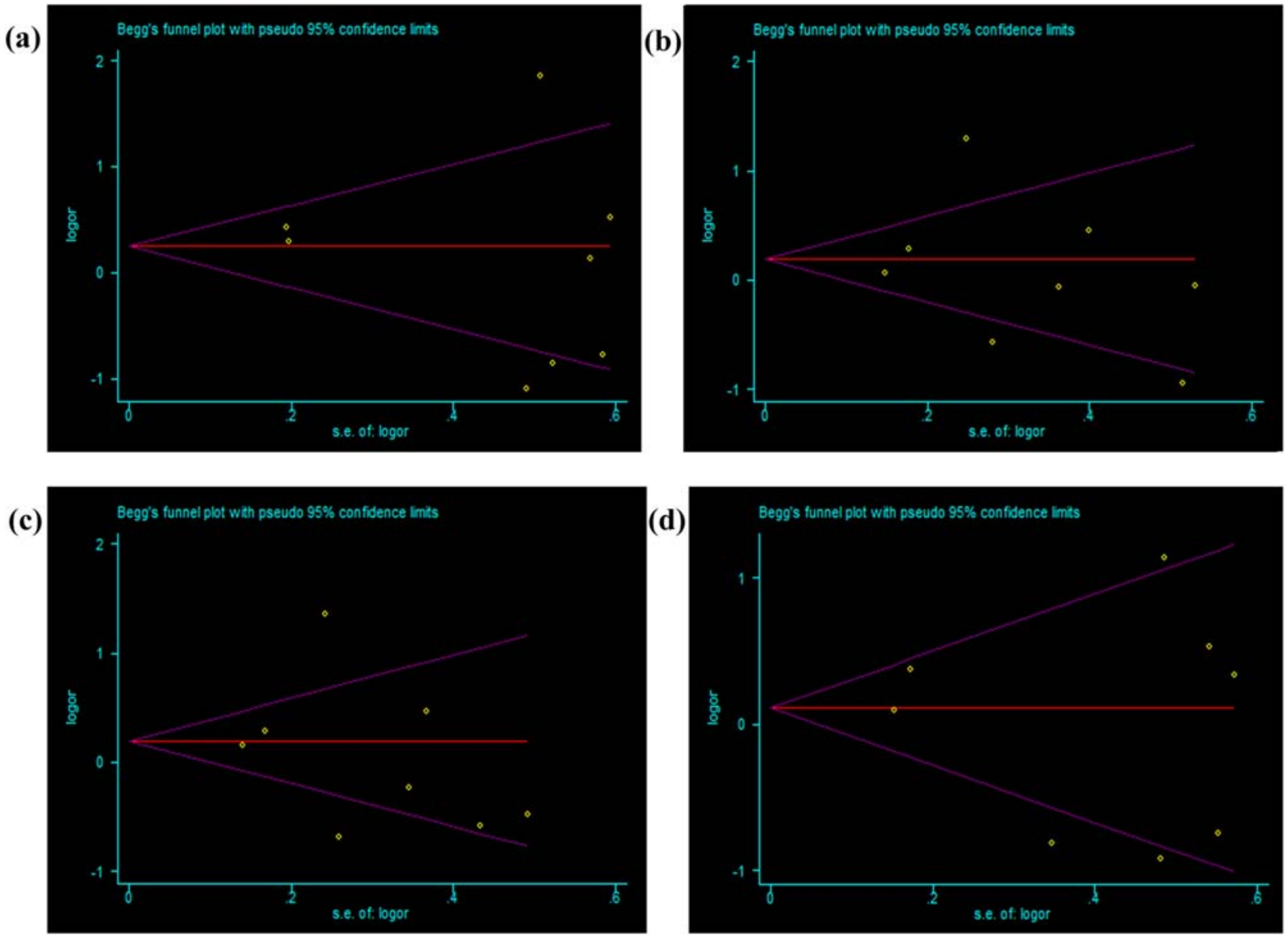

Figure S2: Begg's funnel plots for the relationship between the *IL-10 rs1800872* gene polymorphism and the squamous cell carcinoma risk in four models. A: CC vs. AA; B: AC vs. AA; C: AC+CC vs. AA; D: CC vs. AA+AC.

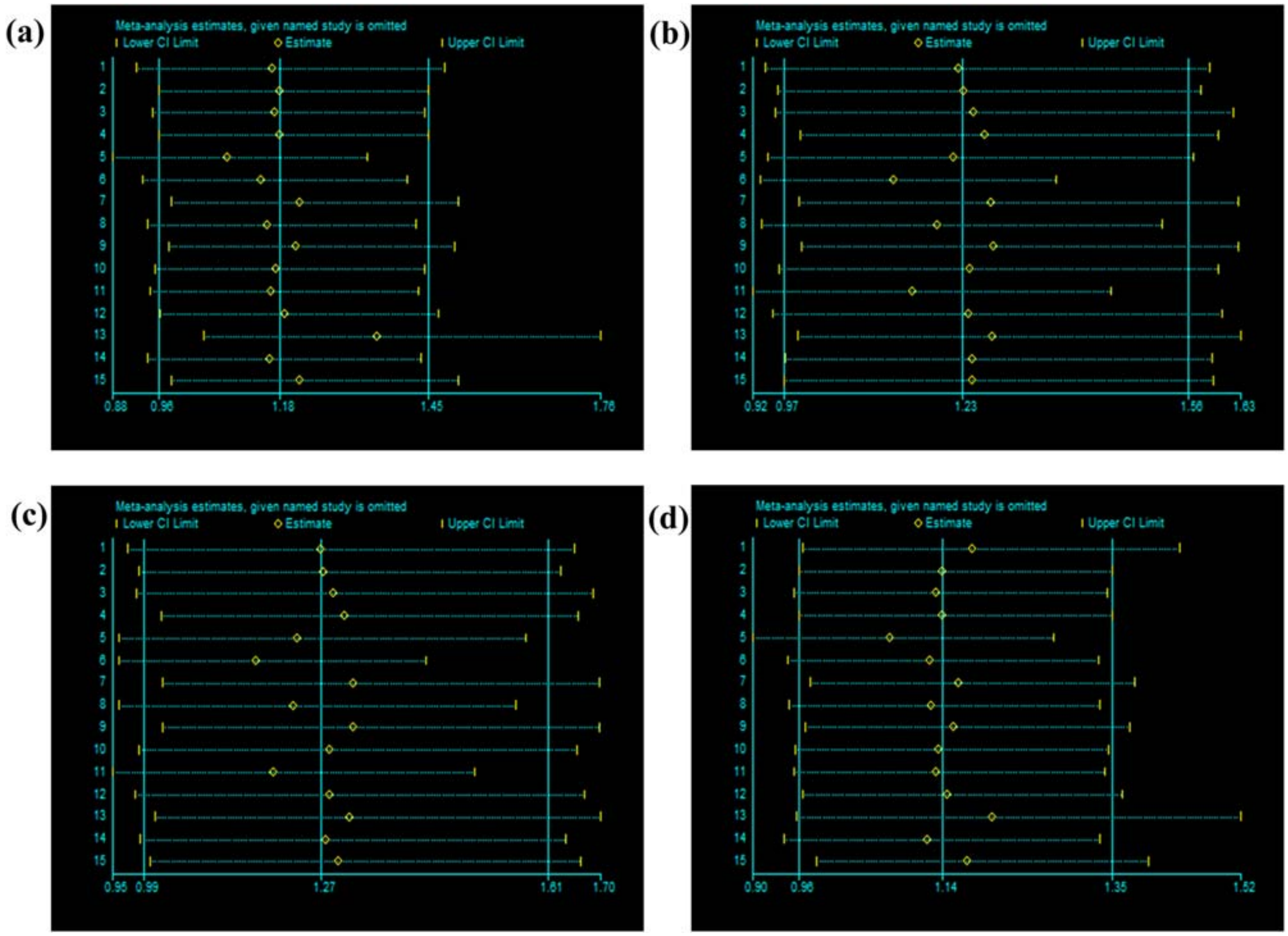

Figure S3: Begg's funnel plots for the relationship between the IL-10 rs1800795 gene polymorphism and the squamous cell carcinoma risk in four models. A: CC vs. GG; B: GC vs. GG; C: GC+CC vs. GG; D: CC vs. GG+GC.

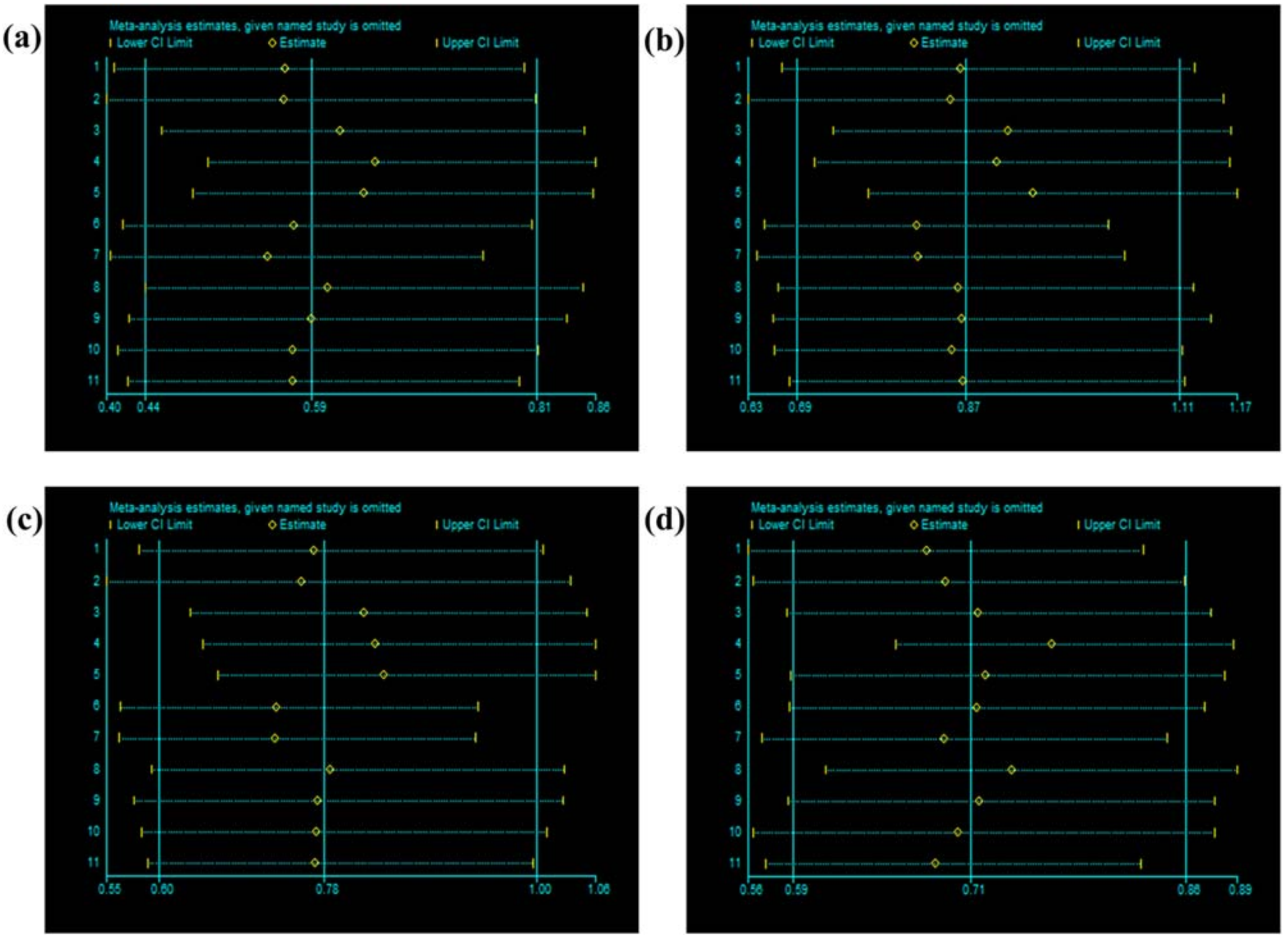

Figure S4: Sensitivity analysis of the association between the *IL-10 rs1800896* gene polymorphism and the squamous cell carcinoma risk in four models. A: GG vs. AA; B: AG vs. AA; C: AG+GG vs. AA; D: GG vs. AA+AG.

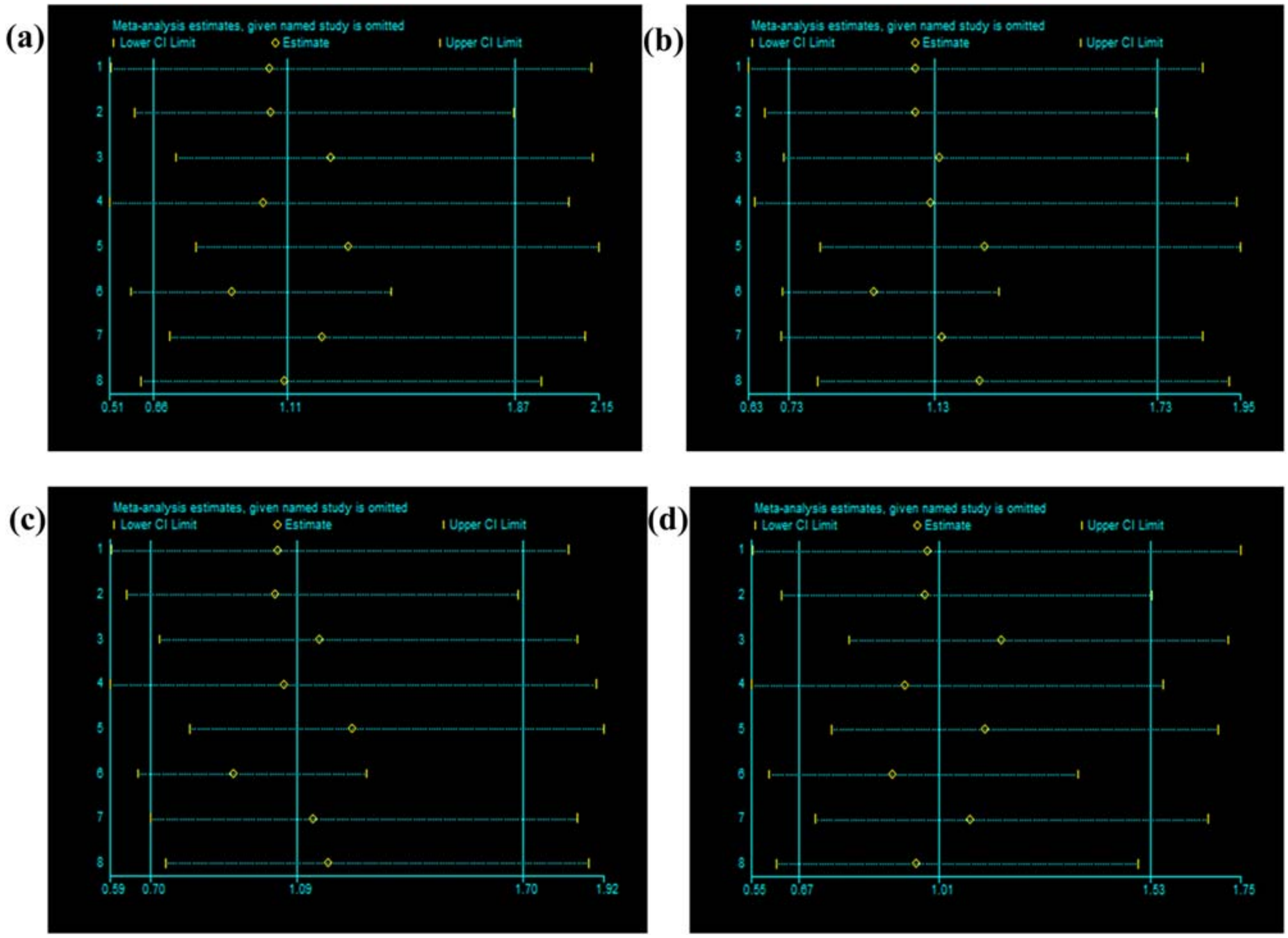

Figure S5: Sensitivity analysis of the association between the *IL-10 rs1800872* gene polymorphism and the squamous cell carcinoma risk in four models. A: CC vs. AA; B: AC vs. AA; C: AC+CC vs. AA; D: CC vs. AA+AC.

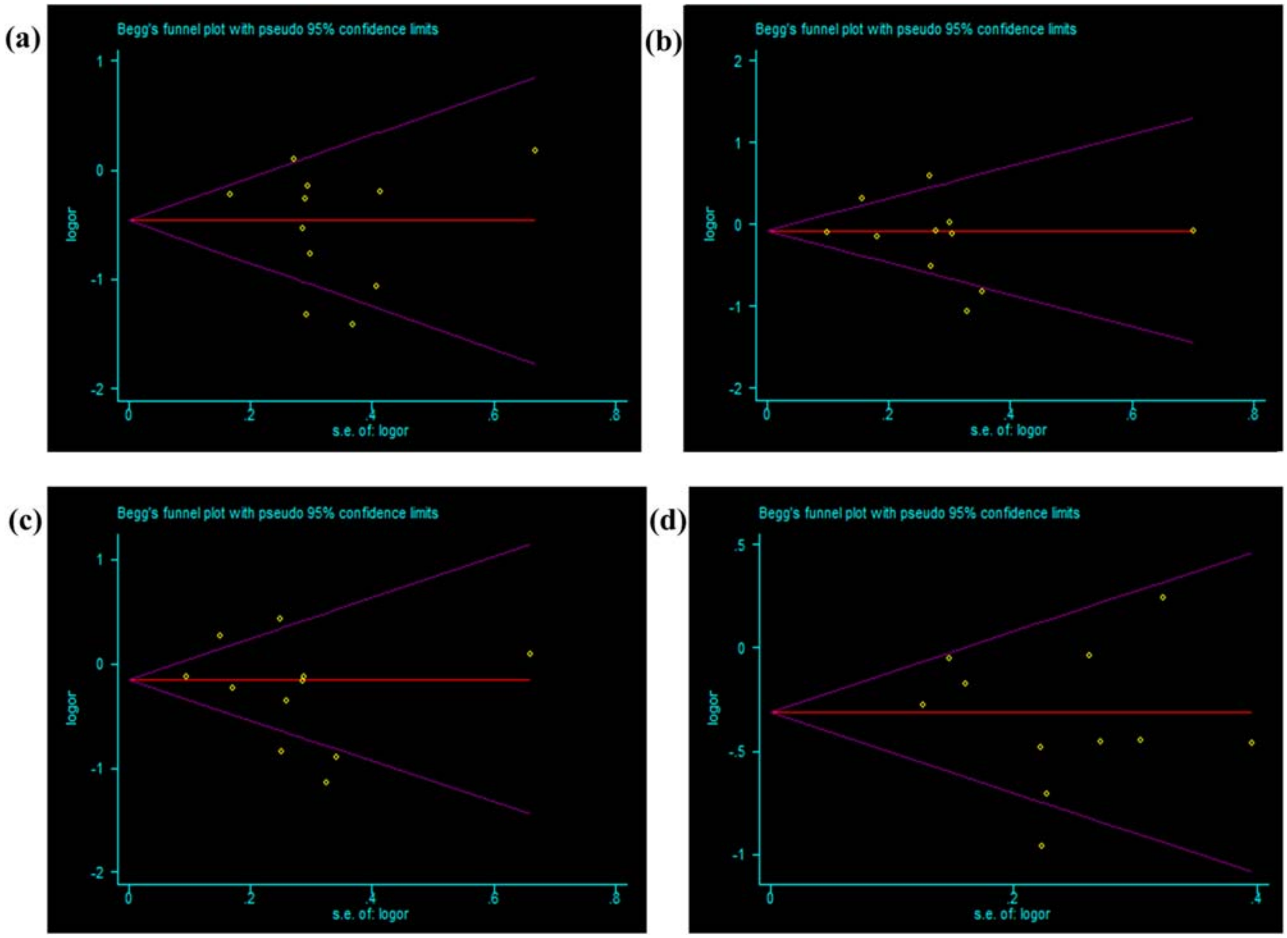

Figure S6: Sensitivity analysis of the association between the *IL-10 rs1800795* gene polymorphism and the squamous cell carcinoma risk in four models. A: CC vs. GG; B: GC vs. GG; C: GC+CC vs. GG; D: CC vs. GG+GC.

Table S1: Meta-regression of the rs1800896, rs1800872 and rs1800795 polymorphisms on squamous cell carcinoma risk

Comparative model	Year		Ethnicity		Source of control	
	t	*p*	*t*	*p*	*t*	*p*
**IL-10 rs1800896**						
**GG/AA**	0.94	0.369	0.67	0.519	−0.69	0.502
**AG/AA**	1.07	0.309	−0.2	0.846	0.22	0.827
**AG+GG/AA**	0.4	0.693	−0.48	0.643	−0.13	0.895
**GG/AA+AG**	1.06	0.311	0.66	0.522	−0.85	0.414
**IL-10 rs1800872**						
**CC/AA**	−0.57	0.584	1.81	0.104	−0.43	0.68
**AC/AA**	−0.56	0.588	2.05	0.071	−0.47	0.649
**AC+CC/AA**	−0.59	0.572	2.00	0.077	−0.44	0.673
**CC/AA+AC**	−0.27	0.792	0.64	0.540	0.22	0.833
**IL-6 rs1800795**						
**CC/GG**	−0.05	0.961	−0.6	0.573	−0.52	0.623
**GC/GG**	−0.03	0.981	−0.68	0.52	−0.44	0.672
**GC+CC/GG**	−0.03	0.979	−0.68	0.521	−0.45	0.668
**CC/GG+GC**	−0.38	0.715	−0.35	0.736	−0.63	0.553

Table S2: Stratified analyses of the IL-10 rs1800896 polymorphism on squamous cell carcinoma risk

Comparative model	FPRP P-value	FPRP Statistical power	FPRP prior probability					BEDP prior probability		
			**0.25**	**0.10**	**0.01**	**0.001**	**0.0001**	**0.010**	**0.001**	**0.000**
**GG/AA**										
**Overall**	**0.110**	0.989	0.250	0.500	0.917	0.991	0.999	0.994	0.999	1.000
**Ethnicity**										
Caucasian	0.470	0.882	0.615	0.828	0.981	0.998	1.000	0.996	1.000	1.000
Asian	0.212	0.449	0.586	0.809	0.979	0.998	1.000	0.991	0.999	1.000
**Source of control**										
HB	**0.176**	0.829	0.389	0.657	0.955	0.995	1.000	0.993	0.999	1.000
PB	0.477	0.781	0.647	0.846	0.984	0.998	1.000	0.995	0.999	1.000
**Cancer types**										
Laryngeal SCC	**0.194**	0.751	0.437	0.700	0.962	0.996	1.000	0.993	0.999	1.000
Oral SCC	**0.000**	**0.000**	0.408	0.674	0.958	0.996	1.000	0.873	0.986	1.000
Cervical SCC	0.460	0.977	0.586	0.809	0.979	0.998	1.000	0.997	1.000	1.000
Esophageal SCC	0.771	0.842	0.733	0.889	0.989	0.999	1.000	0.995	0.999	1.000
**AG/AA**										
**Overall**	**0.094**	0.951	0.228	0.470	0.907	0.990	0.999	0.992	0.999	1.000
**Ethnicity**										
Caucasian	**0.189**	0.806	0.414	0.679	0.959	0.996	1.000	0.993	0.999	1.000
Asian	0.243	0.999	0.422	0.687	0.960	0.996	1.000	0.997	1.000	1.000
**Source of control**										
HB	0.219	0.781	0.457	0.716	0.965	0.996	1.000	0.993	0.999	1.000
PB	0.291	0.932	0.484	0.737	0.969	0.997	1.000	0.995	1.000	1.000
**Cancer types**										
Laryngeal SCC	**0.029**	0.645	**0.120**	0.291	0.818	0.978	0.998	0.976	0.998	1.000
Oral SCC	**0.033**	0.275	0.267	0.523	0.923	0.992	0.999	0.973	0.997	1.000
Cervical SCC	**0.073**	0.974	0.183	0.401	0.881	0.987	0.999	0.991	0.999	1.000
Esophageal SCC	0.513	0.999	0.606	0.822	0.981	0.988	1.000	0.998	1.000	1.000
**AG+GG/AA**										
**Overall**	**0.057**	0.914	**0.157**	0.359	0.860	0.984	0.998	0.988	0.999	1.000
**Ethnicity**										
Caucasian	**0.130**	0.742	0.344	0.612	0.945	0.994	0.999	0.991	0.999	1.000
Asian	**0.160**	0.999	0.325	0.591	0.941	0.994	0.999	0.996	1.000	1.000
**Source of control**										
HB	0.205	0.772	0.443	0.705	0.963	0.996	1.000	0.993	0.999	1.000
PB	**0.057**	0.914	0.157	0.359	0.860	0.984	0.998	0.988	0.999	1.000
**Cancer types**										
Laryngeal SCC	0.802	0.619	0.795	0.921	0.992	0.999	1.000	0.992	0.999	1.000
Oral SCC	**0.015**	0.748	**0.058**	**0.157**	0.672	0.954	0.995	0.964	0.996	1.000
Cervical SCC	0.264	0.998	0.442	0.704	0.963	0.996	1.000	0.997	1.000	1.000
Esophageal SCC	**0.000**	**0.056**	**0.018**	**0.053**	0.381	0.861	0.984	**0.505**	0.911	0.999

(*Continued*)

Table S2: *Continued*

Comparative model	FPRP P-value	FPRP Statistical power	FPRP prior probability					BEDP prior probability		
			**0.25**	**0.10**	**0.01**	**0.001**	**0.0001**	**0.010**	**0.001**	**0.000**
**GG/AA+AG**										
**Overall**	**0.151**	0.999	0.312	0.576	0.937	0.993	0.999	0.996	1.000	1.000
**Ethnicity**										
Caucasian	0.226	0.999	0.404	0.670	0.957	0.996	1.000	0.997	1.000	1.000
Asian	0.264	0.503	0.611	0.825	0.981	0.998	1.000	0.992	0.999	1.000
**Source of control**										
HB	0.546	0.981	0.626	0.834	0.982	0.998	1.000	0.997	1.000	1.000
PB	**0.179**	0.991	0.351	0.619	0.947	0.994	0.999	0.995	1.000	1.000
**Cancer types**										
Laryngeal SCC	0.631	0.972	0.661	0.854	0.985	0.998	1.000	0.997	1.000	1.000
Oral SCC	**0.000**	**0.002**	0.454	0.714	0.965	0.996	1.000	0.923	0.992	1.000
Cervical SCC	0.896	0.999	0.729	0.890	0.989	0.999	1.000	0.998	1.000	1.000
Esophageal SCC	0.610	0.814	0.692	0.871	0.987	0.999	1.000	0.995	0.999	1.000

Abbreviations: PB, Population-based; HB, Hospital-based; SCC, squamous cell carcinoma. FPRP, false positive report probability; BFDP, Bayesian False Discovery Probability; The results in bold represented there was statistically significant noteworthiness at 0.2 level by FPRP or 0.8 level by BFDP calculations.

Table S3: Stratified analyses of the IL-10 rs1800872 polymorphism on squamous cell carcinoma risk

Comparative model	FPRP P-value	FPRP Statistical power	FPRP prior probability					BEDP prior probability		
			0.250	0.10	0.01	0.001	0.0001	0.01	0.001	0.000001
**CC/AA**										
**Overall**	**0.001**	0.232	**0.011**	0.032	0.264	0.783	0.973	**0.677**	0.955	1.000
**Ethnicity**										
Caucasian	**0.001**	**0.068**	**0.035**	**0.097**	0.541	0.923	0.992	**0.676**	0.955	1.000
Asian	**0.076**	0.943	**0.196**	0.422	0.889	0.988	0.999	0.991	0.999	1.000
**Source of control**										
HB	**0.054**	0.418	0.278	0.536	0.927	0.992	0.999	0.981	0.998	1.000
PB	**0.000**	**0.091**	**0.001**	**0.003**	**0.036**	0.276	0.792	**0.114**	**0.566**	0.992
**Cancer types**										
Laryngeal SCC	0.504	0.847	0.641	0.843	0.983	0.998	1.000	0.995	1.000	1.000
Oral SCC	**0.000**	**0.001**	**0.013**	**0.039**	0.310	0.819	0.978	**0.040**	**0.298**	0.977
Cervical SCC	**0.011**	**0.111**	0.229	0.472	0.908	0.990	0.999	0.945	0.994	1.000
Esophageal SCC	**0.102**	0.950	0.244	0.492	0.914	0.991	0.999	0.992	0.999	1.000
**AC/AA**										
**Overall**	0.266	0.985	0.447	0.708	0.964	0.996	1.000	0.996	1.000	1.000
**Ethnicity**										
Caucasian	**0.001**	0.561	**0.006**	**0.019**	**0.173**	0.678	0.955	**0.757**	0.969	1.000
Asian	0.436	0.970	0.574	0.802	0.978	0.998	1.000	0.996	1.000	1.000
**Source of control**										
HB	0.669	0.996	0.668	0.858	0.985	0.999	1.000	0.998	1.000	1.000
PB	0.348	0.756	0.580	0.806	0.979	0.998	1.000	0.994	0.999	1.000
**Cancer types**										
Laryngeal SCC	0.463	0.658	0.678	0.863	0.986	0.999	1.000	0.993	0.999	1.000
Oral SCC	**0.000**	**0.012**	**0.020**	**0.056**	0.397	0.869	0.985	**0.258**	**0.778**	0.997
Cervical SCC	**0.188**	0.882	0.390	0.658	0.955	0.995	1.000	0.994	0.999	1.000
Esophageal SCC	0.873	1.000	0.724	0.887	0.989	0.999	1.000	0.999	1.000	1.000
**AC+CC/AA**										
**Overall**	**0.050**	0.877	**0.146**	0.339	0.850	0.983	0.998	0.986	0.999	1.000
**Ethnicity**										
Caucasian	**0.000**	**0.105**	**0.000**	**0.000**	**0.001**	**0.011**	**0.104**	**0.006**	**0.058**	0.861
Asian	0.717	0.994	0.684	0.867	0.986	0.999	1.000	0.997	1.000	1.000
**Source of control**										
HB	0.241	0.895	0.447	0.708	0.964	0.996	1.000	0.995	0.999	1.000
PB	**0.166**	0.638	0.439	0.701	0.963	0.996	1.000	0.991	0.999	1.000
**Cancer types**										
Laryngeal SCC	0.549	0.804	0.972	0.860	0.985	0.999	1.000	0.995	0.999	1.000
Oral SCC	**0.000**	**0.004**	**0.009**	**0.026**	0.227	0.748	0.967	**0.069**	**0.426**	0.987
Cervical SCC	**0.001**	0.298	**0.014**	**0.041**	0.320	0.826	0.979	0.767	0.971	1.000
Esophageal SCC	0.841	0.999	0.716	0.883	0.988	0.999	1.000	0.998	1.000	1.000

(*Continued*)

Table S3: *Continued*

Comparative model	FPRP P-value	FPRP Statistical power	FPRP prior probability					BEDP prior probability		
			**0.250**	**0.10**	**0.01**	**0.001**	**0.0001**	**0.01**	**0.001**	**0.000001**
**CC/AA+AC**										
**Overall**	**0.000**	0.750	**0.001**	**0.004**	**0.043**	0.314	0.821	**0.539**	0.922	0.999
**Ethnicity**										
Caucasian	**0.004**	0.545	**0.023**	**0.067**	0.440	0.888	0.988	0.900	0.989	1.000
Asian	**0.049**	0.937	**0.136**	0.320	0.838	0.981	0.998	0.987	0.999	1.000
**Source of control**										
HB	**0.036**	0.567	0.161	0.365	0.863	0.985	0.998	0.978	0.998	1.000
PB	**0.000**	0.842	**0.001**	**0.004**	**0.043**	0.314	0.821	**0.575**	0.932	0.999
**Cancer types**										
Laryngeal SCC	0.463	0.987	0.585	0.809	0.979	0.998	1.000	0.997	1.000	1.000
Oral SCC	**0.009**	0.359	**0.068**	**0.179**	0.706	0.960	0.996	0.935	0.993	1.000
Cervical SCC	**0.005**	**0.167**	**0.085**	0.219	0.755	0.969	0.997	0.902	0.989	1.000
Esophageal SCC	**0.182**	0.988	0.355	0.623	0.948	0.995	0.999	0.995	1.000	1.000

Abbreviations: PB, Population-based; HB, Hospital-based; SCC, squamous cell carcinoma. FPRP, false positive report probability; BFDP, Bayesian False Discovery Probability; The results in bold represented there was statistically significant noteworthiness at 0.2 level by FPRP or 0.8 level by BFDP calculations.

Table S4: Stratified analyses of the IL-6 rs1800795 polymorphism on squamous cell carcinoma risk

Comparative model	FPRP P-value	FPRP Statistical power	FPRP prior probability					BEDP prior probability		
			**0.25**	**0.10**	**0.01**	**0.001**	**0.0001**	**0.01**	**0.001**	**0.000001**
**CC/GG**										
**Overall**	0.703	0.873	0.707	0.879	0.988	0.999	1.000	0.995	1.000	1.000
**Ethnicity**										
Caucasian	0.539	0.701	0.698	0.874	0.987	0.999	1.000	0.994	0.999	1.000
Asian	0.717	0.568	0.791	0.919	0.992	0.999	1.000	0.992	0.999	1.000
**Source of control**										
HB	**0.009**	0.630	**0.042**	**0.115**	0.589	0.935	0.993	0.944	0.994	1.000
PB	0.938	0.777	0.784	0.916	0.992	0.999	1.000	0.993	0.999	1.000
**Cancer types**										
Oral SCC	0.974	0.653	0.817	0.931	0.993	0.999	1.000	0.991	0.999	1.000
Laryngeal SCC	**0.086**	0.677	0.277	0.535	0.927	0.992	0.999	0.988	0.999	1.000
**GC/GG**										
**Overall**	0.585	0.904	0.660	0.853	0.985	0.998	1.000	0.996	1.000	1.000
**Ethnicity**										
Caucasian	0.387	0.708	0.621	0.831	0.982	0.998	1.000	0.994	0.999	1.000
Asian	0.516	0.736	0.678	0.863	0.986	0.999	1.000	0.994	0.999	1.000
**Source of control**										
HB	**0.158**	0.984	0.325	0.591	0.941	0.994	0.999	0.995	0.999	1.000
PB	0.880	0.820	0.763	0.906	0.990	0.999	1.000	0.997	1.000	1.000
**Cancer types**										
Oral SCC	0.719	0.594	0.784	0.916	0.992	0.999	1.000	0.992	0.999	1.000
Laryngeal SCC	**0.052**	0.710	**0.179**	0.396	0.878	0.986	0.999	0.984	0.998	1.000
**GC+CC/GG**										
**Overall**	0.697	0.920	0.694	0.872	0.989	0.999	1.000	0.996	1.000	1.000
**Ethnicity**										
Caucasian	0.494	0.753	0.663	0.855	0.985	0.998	1.000	0.994	0.999	1.000
Asian	0.697	0.920	0.694	0.872	0.987	0.999	1.000	0.996	1.000	1.000
**Source of control**										
HB	**0.045**	0.961	**0.122**	0.294	0.821	0.979	0.998	0.987	0.999	1.000
PB	0.996	0.846	0.779	0.914	0.991	0.999	1.000	0.994	0.999	1.000
**Cancer types**										
Oral SCC	0.906	0.663	0.804	0.925	0.993	0.999	1.000	0.992	0.999	1.000
Laryngeal SCC	**0.034**	0.708	**0.127**	0.304	0.828	0.980	0.998	0.979	0.998	1.000
**CC/GG+GC**										
**Overall**	0.959	0.969	0.748	0.899	0.990	0.999	1.000	0.996	1.000	1.000
**Ethnicity**										
Caucasian	0.846	0.899	0.738	0.894	0.989	0.999	1.000	0.995	1.000	1.000
Asian	0.766	0.629	0.785	0.916	0.992	0.999	1.000	0.992	0.999	1.000

(*Continued*)

Table S4: *Continued*

Comparative model	FPRP P-value	FPRP Statistical power	FPRP prior probability					BEDP prior probability		
			**0.25**	**0.10**	**0.01**	**0.001**	**0.0001**	**0.01**	**0.001**	**0.000001**
**Source of control**										
HB	**0.053**	0.948	**0.143**	0.334	0.846	0.982	0.998	0.988	0.999	1.000
PB	0.769	0.795	0.744	0.897	0.990	0.999	1.000	0.994	0.999	1.000
**Cancer types**										
Oral SCC	0.727	0.615	0.780	0.914	0.992	0.999	1.000	0.992	0.999	1.000
Laryngeal SCC	0.441	0.977	0.575	0.802	0.978	0.998	1.000	0.997	1.000	1.000

Abbreviations: PB, Population-based; HB, Hospital-based; SCC, squamous cell carcinoma. FPRP, false positive report probability; BFDP, Bayesian False Discovery Probability; The results in bold represented there was statistically significant noteworthiness at 0.2 level by FPRP or 0.8 level by BFDP calculations.
